# Uncovering the biosynthetic potential of rare metagenomic DNA using co-occurrence network analysis of targeted sequences

**DOI:** 10.1038/s41467-019-11658-z

**Published:** 2019-08-26

**Authors:** Vincent Libis, Niv Antonovsky, Mengyin Zhang, Zhuo Shang, Daniel Montiel, Jeffrey Maniko, Melinda A. Ternei, Paula Y. Calle, Christophe Lemetre, Jeremy G. Owen, Sean F. Brady

**Affiliations:** Laboratory of Genetically Encoded Small Molecules, The Rockefeller University, 1230 York Avenue, New York, NY 10065 USA

**Keywords:** Soil microbiology, Natural products, Metagenomics

## Abstract

Sequencing of DNA extracted from environmental samples can provide key insights into the biosynthetic potential of uncultured bacteria. However, the high complexity of soil metagenomes, which can contain thousands of bacterial species per gram of soil, imposes significant challenges to explore secondary metabolites potentially produced by rare members of the soil microbiome. Here, we develop a targeted sequencing workflow termed CONKAT-seq (co-occurrence network analysis of targeted sequences) that detects physically clustered biosynthetic domains, a hallmark of bacterial secondary metabolism. Following targeted amplification of conserved biosynthetic domains in a highly partitioned metagenomic library, CONKAT-seq evaluates amplicon co-occurrence patterns across library subpools to identify chromosomally clustered domains. We show that a single soil sample can contain more than a thousand uncharacterized biosynthetic gene clusters, most of which originate from low frequency genomes which are practically inaccessible through untargeted sequencing. CONKAT-seq allows scalable exploration of largely untapped biosynthetic diversity across multiple soils, and can guide the discovery of novel secondary metabolites from rare members of the soil microbiome.

## Abstract (web summary)

Soil microorganisms are a rich source of bioactive molecules. Here, the authors present a targeted sequencing workflow that reconstructs the clustered organization of biosynthetic domains in metagenomic libraries from amplicon data, thus guiding the discovery of novel metabolites from rare members of the soil microbiome.

## Introduction

**Fig. 1:**
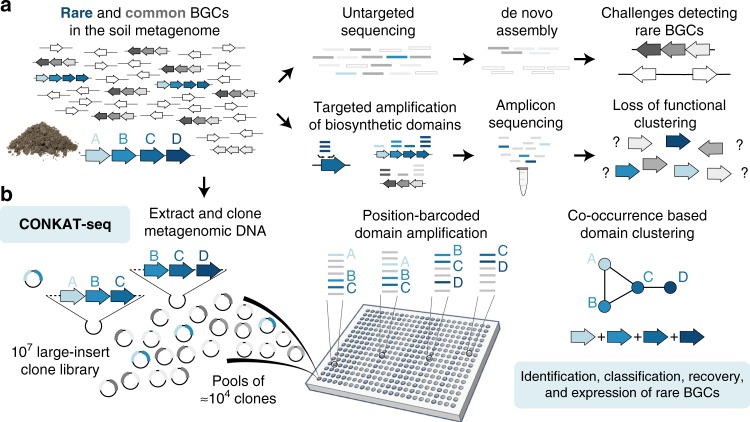
CONKAT-seq enables the exploration of rare biosynthetic gene clusters in complex metagenomes. **a** Untargeted methods to explore the biosynthetic potential of low frequency organisms in the soil metagenome are limited by the required high coverage depth, and computationally challenging de novo assembly process. PCR based methods are extremely sensitive, but do not capture the functional clustering of biosynthetic domain and therefore are information poor. **b** CONKAT-seq uses the highly partitioned structure of metagenomic cosmid libraries to reconstruct the chromosomal organization of biosynthetic domains based on PCR amplicon data

Secondary metabolites produced by soil dwelling microorganisms have been a rich source of bioactive molecules, with applications in diverse therapeutic areas. Unfortunately, the vast majority of environmental bacteria remain recalcitrant to culture in the laboratory and therefore cannot be studied using culture-based methods. In bacterial genomes, pathways for the production of secondary metabolites are typically encoded in clusters of physically adjacent genes, known as biosynthetic gene clusters (BGCs). Although untargeted sequencing has been used to identify novel BGCs in soil metagenomes, these efforts face steep diminishing returns due to the skewed distribution of species abundance in the soil^1^. As such, although low abundance species represent the majority of biodiversity^2,3^, little is known about the biosynthetic potential of these organisms. An alternative approach to explore biosynthetic diversity in the soil metagenome relies on the amplification and phylogenetic analysis of conserved protein domains in biosynthetic genes (“biosynthetic domains”), similar to the use of 16S surveys to measure species diversity^4^. Due to the ease with which amplicons can be deeply sequenced, this method provides ready access to diverse biosynthetic domain sequences present in metagenomes^5^. However, despite the unparalleled sensitivity of PCR based experiments, critical information regarding the clustering of biosynthetic domains into pathways is inherently lost in the resulting heterogeneous mixture of single domain amplicons (Fig. 1a).

To address this shortcoming and enable a more informative exploration of rare BGCs in soil metagenomes, we sought to develop a sequencing workflow that would reconstruct the chromosomal organization of biosynthetic domains into clusters from amplicon sequencing data.

## Results

### Development of CONKAT-seq

We reasoned that capturing environmental DNA in a clone library would facilitate the sequencing of BGCs present in low frequencies in the metagenome. Cloning the massive genetic diversity present in most soil samples requires the construction of libraries containing millions of cosmid clones harboring metagenomic DNA inserts^6^. Libraries of this size are typically arrayed in subpools, each containing several thousand unique clones. As the large (≈40 kbp) fragment of metagenomic DNA captured in each library clone preserves the chromosomal organization of genes, we reasoned that amplicons originating from clustered genes would show high levels of co-occurrence across subpools due to their high probability of being co-captured (Fig. 1b). CONKAT-seq relies on the statistical analysis of amplicon co-occurrence across hundreds of library subpools to identify networks of physically clustered biosynthetic domains, and uses these predictions to point towards novel BGCs encoded in a metagenome (Supplementary Fig. 1). First, biosynthetic domains of interest are amplified from the library using degenerate primer pairs containing subpool-specific barcodes. The resulting amplicons are sequenced and debarcoded so that each domain variant is traced to the library subpool from which it was amplified. We then determine the pairwise co-occurrence frequencies of biosynthetic domain variants across all subpools and use Fisher’s exact test to identify domain pairs that show strong linkage. Finally, CONKAT-seq predictions are visualized as networks, where nodes represent sequence variants of the targeted biosynthetic domains and edges link domains predicted to be physically co-clustered in the metagenomic DNA. Although many BGCs are larger than the size that can be captured on a single cosmid insert, this does not limit our analysis as these BGCs are often captured in multiple, partially overlapping clones from which CONKAT-seq can generate linkage networks (Fig. 1b).

### Domain networks faithfully predict BGCs and their novelty

**Fig. 2:**
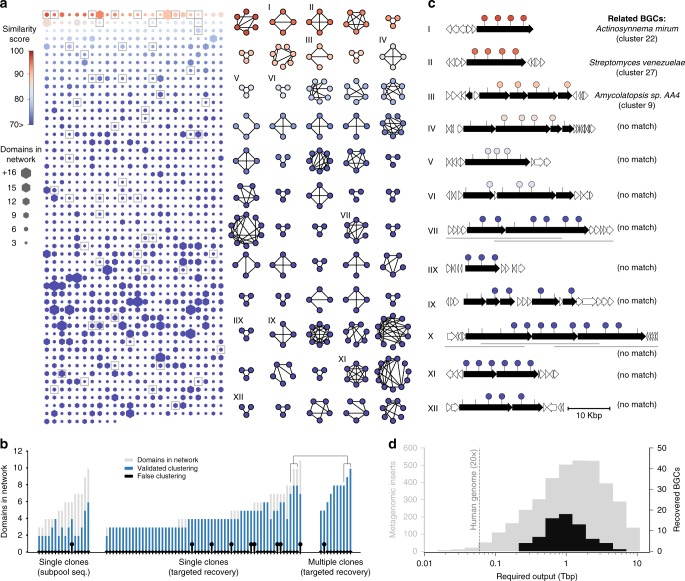
A single metagenome can contain hundreds of previously uncharacterized, low-frequency BGCs that are practically inaccessible through untargeted sequencing. **a** Visualization of clustered domain networks predicted by using CONKAT-seq on a soil metagenomic library. Hexagons represent networks with size proportional to the number of domains and colored according to their similarity score to known BGCs. Only networks with between 3 and 30 domains are presented. A subset of 60 networks (squares) is presented in detail where nodes correspond to AD and KS domain variants and edges link variants that are predicted to be physically co-clustered. Nodes are colored based on the similarity score of the network. **b** Domain network predictions were validated using long-read sequencing of library subpools or by the recovery and sequencing of library clones encoding BGCs. Each bar is associated with a specific domain network (Supplementary Data 1). Based on assembled contigs from sequenced subpools (left) or recovered clones (middle and right) the number of experimentally validated domain clustering predictions is presented (blue) in comparison to the total number of domains in the network (gray). Black bars represent the number of false clustering predictions (i.e., domains in the network that were not present in the metagenomic insert). In cases where the length of BGCs exceeds the size of the metagenomic insert, only a subset of the domains could be validated from a single cosmid (middle, gray vs. blue bars). We demonstrate that in such cases all of the domain network can be recovered from multiple overlapping clones (right). **c** Sample of 12 annotated BGCs that were recovered in based on CONKAT-seq predictions. For each BGC we indicate the “closest” BGC based on Bigscape analysis (Supplementary Table 3, Supplementary Fig. 4). Ticks represent actual domains positions and circle markers indicate the domains predicted by the network. Large BGCs that were recovered from multiple overlapping clones (VII and IX) are marked by underlines. **d** Sequencing output (in terabases) required to reach a depth of coverage of 20X of the recovered BGCs (black) and of ≈3000 representative metagenomic inserts found in two subpools of the library (gray)

CONKAT-seq can reconstruct the chromosomal clustering for any collection of two or more conserved genes targeted by degenerate primers. We chose to focus our initial exploration on nonribosomal peptide synthetase (NRPS) and polyketide synthase (PKS) biosynthesis. These gene cluster families are particularly well suited for this type of analysis as they are composed of highly conserved repeating enzymatic domains. As a result, in each case a single set of degenerate primers is sufficient to target multiple domains in a BGC, thereby technically simplifying the generation of data used by CONKAT-seq. The first metagenome we explored using CONKAT-seq was that of an arid soil sample collected in Arizona (Supplementary Table 1). DNA from this soil was captured in a ≈10-million membered cosmid library hosted in *Escherichia coli* (≈38 kb average insert). The library was arrayed as 2304 subpools containing ≈5000 cosmids each. To identify cloned BGCs, we used barcoded degenerate primers to amplify NRPS adenylation (AD) or PKS ketosynthase (KS) domain sequences from all library subpools (Supplementary Table 2). Amplicons derived from these domains range from 500 to 800 bps in size. Sequencing of the amplicon products found an average of 120 AD and KS domain variants in each subpool, and ≈10^5^ unique domain variants in total (Supplementary Fig. 2). By statistically analyzing the co-occurrence frequencies of domain variants across subpools, we identified 13305 domain pairs that show strong linkage. Based on this pairwise analysis we resolved the collection of amplicons into 1233 discrete networks composed of 3 or more domain variants that are predicted to be chromosomally co-clustered in the metagenome. The large number of co-clustered domain networks identified in our analysis  suggests the existence of hundreds of NRPS and PKS BGCs in this metagenomic library. We visualized these results by constructing a graph representation where each network corresponds to a set of biosynthetic domains present in a BGC (Fig. 2a and Supplementary Fig. 3). To validate our CONKAT-seq domain network predictions we sequenced the metagenomic DNA captured in two library subpools (≈0.1% of total library clones) using single-molecule long-read technology. In contigs assembled from this data we were able to validate >98% of the 53 relevant CONKAT-seq domain clustering predictions derived from these subpools (Fig. 2b, left and Supplementary Data 1).

Next, we sought to determine the proportion of domain networks that do not correspond to previously described BGCs, and therefore potentially encode novel secondary metabolites. We compared all of the predicted domain networks in this sample to sequences of NRPS and PKS gene clusters reported in publicly available databases (antiSMASH^7^, 10733 sequences) and to a curated collection of functionally characterized BGCs (MIBiG^8^, 1067 sequences). We considered a domain network to be affiliated with a reference BGC if half or more of its domains matched those of a reference with 75% or greater amino acid identity (Fig. 2a, c, Methods). Similar metrics comparing shared biosynthetic domains have been employed to assess relationships between fully sequenced BGCs^9,10^. Our analysis found that only 14% of networks were related to BGCs in sequenced genomes, and only 5% of these were related to a BGC whose products have been identified (Fig. 2a).

A significant advantage of cloned metagenomic DNA libraries is that the genetic diversity present in the sample is stably captured and is therefore readily accessible. As CONKAT-seq was performed solely on the targeted NRPS and PKS domains, our analysis does not yield comprehensive information regarding the diverse tailoring genes that could be present in BGCs. However, once BGCs of interest have been identified based on CONKAT-seq predictions, clones containing these BGCs can be recovered from the library subpools, and sequenced to yield the full composition of the cluster. In brief, each domain network specifies a set of domain sequences and the subpools in which these were detected. CONKAT-seq predictions can therefore guide the recovery of specific BGC encoding clones from the library using a serial dilution PCR strategy (Methods). To test CONKAT-seq predictions, we recovered and sequenced 60 metagenomic clones associated with domain networks classified by our metric as either novel (*n* = 44) or related to a previously sequenced BGC (*n* = 16; Supplementary Fig. 4). Using software tools designed to classify and compare BGCs (BiG-SCAPE^11^ and CORASON^11^) we investigated the metagenomic BGCs found in the clones we recovered. For 55 clones (91%) this analysis recapitulated the classification predicted using CONKAT-seq networks (Fig. 2c and Supplementary Table 3). For these 55 positive examples, clones associated with CONKAT-seq networks that we predicted to be novel were not bioinformatically classified as being part of any existing BGC family, while those predicted to be associated with a known BGC family, showed high similarity to BGCs in the predicted family based on their gene content, order, and sequence identity^11^. In five cases (9%) our metric classified a network as related to a known BGC while the analysis of the full sequence did not match a known family, suggesting our metric slightly underestimates the proportion of novel BGCs in a sample (Supplementary Table 3). Overall, these results suggest that the vast majority (>85%) of CONKAT-seq networks predicted from this soil sample arise from previously unexplored BGCs.

**Fig. 3:**
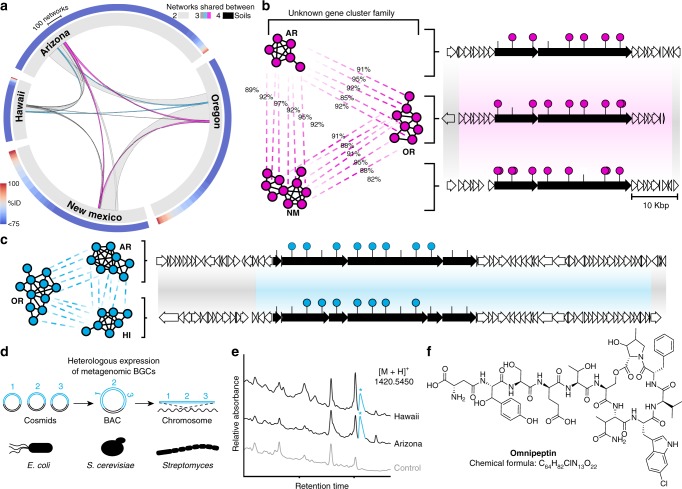
Comparing domain networks from different samples enables discovery of common yet unknown families of natural products. **a** 3591 domain networks obtained from four soil samples were compared to each other and to sequences of BGCs found in databases. For each soil sample, the outer ring (red and blue) depicts the proportions of networks that display similarity to sequences of BGC found in databases. The fraction of networks found in each soil sharing relatives with a median identity higher than 90% in other soil samples is represented by the ratio of the width of the ribbons relative to the corresponding part of the inner ring (gray). Each ribbon represents the fraction of networks shared by combinations of 2, 3, or all 4 soils analyzed. Colored ribbons represent networks with close relatives in Oregon, Arizona and New Mexico (Magenta) or Hawaii, Arizona and Oregon (Cyan) out of which one representative family was physically recovered and depicted in **b** and **c**. **b** Detailed view of the pairwise comparison of biosynthetic domains belonging to related networks that form a family of common yet unknown BGCs. Recovery of metagenomic clones encoding this BGC family from geographically distant soils revealed novel and highly similar gene content and architecture. Colored shading covers the portion of metagenomic DNA showing high homology between related BGCs while gray highlight genes lacking significant homology that likely belong to independent chromosomal contexts outside of BGC boundaries. **c** CONKAT-seq networks and BGCs for members of a family of common yet unknown BGCs identified in Hawaii and Arizona soil samples. **d** The corresponding overlapping cosmids isolated from *E. coli* clones were assembled by transformation-associated recombination in *S. cerevisiae* into a bacterial artificial chromosome (BAC) which was later integrated in the chromosome of *Streptomyces albus*. **e** Heterologous expression of the two related metagenomic BGCs and analysis of the associated crude extracts by HPLC/MS led to the detection of new peaks normally absent in the control extract obtained from the host native background (*S. albus)*. **f** Both BGCs led to the isolation of the same major product, omnipeptin, a novel 11 residue cyclic depsipeptide

**Fig. 4:**
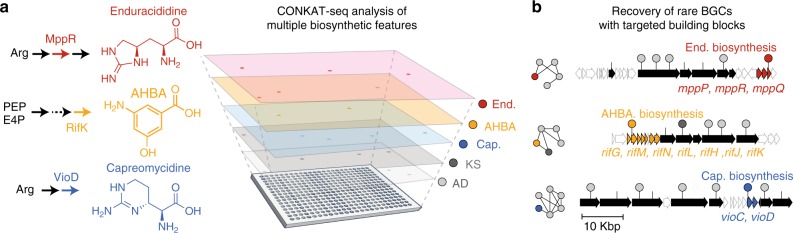
CONKAT-seq identifies BGCs encoding for small molecules with desired chemical features in the soil metagenome. **a** Using a collection of two or more conserved genes targeted by degenerate primers CONKAT-seq reconstructs the chromosomal association of multiple biosynthetic domains. We designed three primer pairs and performed PCR amplification of conserved enzymes in the biosynthesis of molecular building blocks for secondary metabolites: MppR (enduracididine), VioD (capreomycidine), and RifK (3-amino-5-hydroxybenzoic acid, AHBA). By analyzing the co-occurrences frequencies of the resulting amplicons with amplicons of other biosynthetic domains (e.g., AD or KS domains), CONKAT-seq identifies BGCs that specifically incorporate these molecular building blocks in their molecular product. **b** Examples of three BGCs that were recovered from the soil metagenome based on CONKAT-seq predictions

Out of the 245 domains CONKAT-seq predicted to be clustered in the 60 clones we recovered, over 95% (*n* = 237) were found on the metagenomic DNA inserts (Fig. 2b, center and Supplementary Data 1) further validating the accuracy of CONKAT-seq domain network predictions. In cases where a BGC is larger than 40 kbp, the predicted network should include more domains than are present in any individual metagenomic clone. The domains present in CONKAT-seq networks that did not correspond to domains annotated in the 60 recovered clones (Fig. 2b, gray bars) were therefore expected to be present in overlapping clones elsewhere in the library. Over the course of this study, this was tested on 10 networks for which we recovered multiple overlapping clones (Fig. 2c VII and X, Figs. 3 and 4). Out of the 74 domains predicted in these 10 networks, 73 were present in the assembled full BGC sequences (Fig. 2b, right). These results demonstrate the ability of CONKAT-seq to faithfully identify cloned metagenomic DNA encoding multiple biosynthetic domains, predict the novelty of BGCs associated with domain networks and guide the physical recovery of BGCs of interest.

### CONKAT-seq enables the detection of extremely rare BGCs

To estimate the contribution of low abundance species to the biosynthetic diversity detected by CONKAT-seq, we used short read sequencing technology to obtain low-depth coverage of the complete metagenomic library. The resulting reads were mapped to the metagenomic inserts in clones that we recovered based on CONKAT-seq predictions, and the depth of coverage was used as a metric to determine the relative abundance of these BGCs in the library. Our analysis found that the majority of these BGCs originate from genomes present at low frequencies (<0.05%, see Source Data) in the metagenomic library. The discovery of BGCs from such low frequency genomes based on untargeted sequencing and de novo assembly would require a sequencing depth that far exceeds previous efforts for a single soil metagenome^12,13^. As shown in Fig. 2d, BGCs recovered based on CONKAT-seq predictions would require on average the sequencing of more than 1.25 Tbp to obtain even the minimum depth of coverage (20×) required to enable their potential de novo assembly. Although some bias may arise during the isolation and cloning of metagenomic DNA, our analysis suggests that the majority of NRPS and PKS biosynthetic diversity in the soil originates from low frequency bacterial genomes. When taken together with the large number of uncharacterized domain networks identified in our analysis, these results suggest that rare metagenomic DNA contains a rich reservoir of biosynthetic potential that has so far not been explored in conventional untargeted sequencing campaigns.

### Analysis of multiple soils detects common yet unknown BGCs families

To explore the utility of CONKAT-seq for surveying natural product biosynthetic diversity in different environments, we used it to study libraries constructed from soils collected at three additional geographically distinct sites (Hawaii, Oregon, New Mexico). This extended our analysis to a total of 3 Tbp of cloned metagenomic DNA (Supplementary Table 1). The new samples yielded 2329 additional CONKAT-seq domain networks (≥3 domains, Supplementary Fig. 5). The vast majority of these networks showed no significant similarity to any previously described BGCs (Fig. 3a, outer ring). To compare domain networks derived from different soils we used a simple similarity metric, which defined networks as closely related if at least 50% of their domains shared 90% or greater identity. Based on this metric, only a small subset of networks is shared between any two soils (Fig. 3a). Interestingly, the majority (66%) of “common” networks (i.e., networks sharing close relatives in multiple soil samples), were absent from publicly available databases (no relatives with a median domain identity of >75%), suggesting that even common families of BGCs in the soil have evaded conventional discovery approaches.

To further study these geographically common yet uncharacterized BGC families, we used our similarity analysis to guide the recovery and sequencing of metagenomic clones from multiple environments that showed high similarity to domain networks of BGCs previously identified in the Arizona library. In agreement with CONKAT-seq predictions, these newly recovered BGCs closely resembled, both by gene content and organization, the BGCs from the Arizona library (Fig. 3b, c). Despite the occurrence of these BGCs in multiple soil samples, we found no examples of these BGCs in databases. Our domain-network based similarity analysis identified examples of sequence conserved BGC families, present in multiple distinct geographical locations, that are so far not found in BGC sequence databases. Such BGCs are appealing for further characterization through heterologous expression of metagenomic DNA as their diverse geographic distribution may suggest a conserved ecological role, and because they do not contain homologs in sequenced bacteria. Heterologous expression of two highly similar BGCs (Fig. 3c) recovered from the Hawaii and Arizona libraries led to the production of the same metabolite by *Streptomyces albus* (Fig. 3d). This novel natural product, which we have termed omnipeptin, is an 11 residue depsipeptide containing four non-proteinogenic residues: β-hydroxy-tyrosine, β-methyl-asparagine, 6-chlorine-tryptophan, β-hydroxy-γ-methyl-proline (Fig. 3b, Supplementary Figs. 6–19, Supplementary Tables 4 and 5). Interestingly, to the best of our knowledge this is the first report of a bacterial NRPS product containing either β-methyl-asparagine or β-hydroxy-γ-methyl-proline. These residues have only been observed previously in nonribosomal peptides isolated from sponges and fungi, respectively. The discovery of omnipeptin suggests that CONKAT-seq networks will be useful for predicting not only BGC sequence novelty but also the potential for BGCs to encode novel metabolites. Based on the low-depth sequencing of the Arizona library we estimate that the omnipeptin BGC would require ≈1 Tbp of sequence to be detected using an untargeted sequencing approach. It is therefore unlikely that this BGC could have been detected in multiple independent samples and as such, prioritized as a common yet unknown family for heterologous expression studies without the use of CONKAT-seq. More generally, our analysis indicates that the soils we examined contain largely nonoverlapping collections of novel BGCs, and that even in the cases where BGCs are common in the environment, the metabolites they encode have potentially not yet been identified using other natural product discovery methods.

### Analysis of multiple domains increases predictive capabilities

CONKAT-seq is not limited to the detection of NRPS/PKS core domains. It can be used to identify the physical association between any collection of conserved target genes. Following the design of suitable degenerate primer pairs for a domain of interest, barcoded primers are used to amplify the target domain across all library subpools. Barcoded amplicons are sequenced to yield information regarding the presence of target domain variants within the library subpools. This process can be repeated for multiple domains of interest, yielding information on the presence of these domains of interest in the library subpools. Co-occurrence analysis is then performed across all of the amplified domains to identify BGC encoding clones that contain more than one type of target domain (Fig. 4). To demonstrate the versatility of CONKAT-seq we targeted the recovery of BGCs that are predicted to incorporate one of three unique building blocks into their biosynthetic product: enduracididine, capreomycidine, 3-amino-5-hydroxybenzoic acid (AHBA) (Fig. 4a). We designed domain specific degenerate primers that recognize conserved regions in genes encoding the biosynthesis of each targeted building block (Supplementary Table 2). We used subpool-barcoded primers to amplify these targets from subpools of the soil libraries (Supplementary Table 1). Using CONKAT-seq, the data from sequenced “building block” amplicons were analyzed together with the previously described AD and KS amplicon datasets. As shown in Fig. 4, our analysis yielded multiple domain networks that include the targeted “building block” domain nodes. Recovery and sequencing of clones associated with these networks identified BGCs that include the genes required for the production of the targeted building blocks. All of the BGCs that we identified using this multiplexed CONKAT-seq approach differ by biosynthetic gene content from any previously sequenced BGCs and are predicted to encode new molecular products that incorporate the target building block. Of particular interest is the first example of a BGC that contains atypical clustering of AHBA biosynthesis genes with multiple AD domains (Fig. 4b). AHBA biosynthesis has typically been found associated with PKS modules^14^. The expansion of CONKAT-seq to a multi-domain format will facilitate the search for novel secondary metabolites with desired chemical features and can be applied to the analysis of BGC families beyond modular NRPS and PKS gene clusters.

## Discussion

Our CONKAT-seq analysis suggests that a large fraction of the biosynthetic diversity in soil metagenomes is encoded by low frequency BGCs that have so-far remained inaccessible to untargeted sequencing efforts. CONKAT-seq relies on the targeted amplification of conserved biosynthetic domains and leverages the modular nature of BGCs to allow large-scale classification and comparative analysis of biosynthetic diversity. We note that due to domain amplification biases CONKAT-seq does not comprehensively capture the biosynthetic diversity within a sample. Even with this limitation, this study demonstrates that CONKAT-seq can identify an extremely large collection of uncharacterized BGCs in the soil metagenome. The diversity of BGCs accessible by CONKAT-seq can be easily scaled using primers targeting either different degenerate sequences or other conserved biosynthetic domains of interest. As CONKAT-seq requires only limited sequencing resources it offers a scalable, economical and computationally simple solution to accelerate the characterization of microbial biosynthetic diversity in the global microbiome especially in the genomes of low frequency and uncultured organisms. We expect CONKAT-seq will be particularly useful for guiding the discovery of novel natural products from the largely untapped reservoir of biosynthetic potential encoded in rare bacteria.

## Methods

### Metagenomic library construction

Detailed protocol for library construction can be found in Brady et. al.^6^. In brief, ≈250 g of soil were collected from each sample site (Supplementary Table 1). Soil was sifted to remove large particulates, heated to 70 °C for 2 h in lysis buffer [100 mM Tris-HCl, 100 mM EDTA, 1.5 M NaCl, 1% (wt/vol) CTAB (cetyltrimethylammonium bromide), 2% (wt/vol) SDS, pH 8.0], and centrifuged to remove particulates from the crude lysate. DNA was precipitated by the addition of 70% isopropanol (vol/vol), collected by centrifugation, washed with 70% (vol/vol) ethanol, and resuspended in Tris/EDTA buffer [10 mM Tris, 1 mM EDTA, pH 8.0]. Gel-purified (1% agarose) high-molecular-weight DNA was blunt-ended (End-It, Epicentre), and ligated into pWEB-TNC (Epicentre). The metagenomic cosmid library was packaged into lambda phage (MaxPlax, Epicentre), and transfected into *Escherichia coli* EC100 to yield ≈5000 transfected cells per each subpool (as measured by CFU on selective media). Matching glycerol stocks and cosmid DNA minipreps (normalized to 100 ng/μl) were prepared from each subpool and arrayed in 384-well plates for downstream CONKAT-seq analysis. Typically, the construction and arraying of a cosmid metagenomic library into subpools takes ≈2 weeks. Glycerol stock cultures from library subpools were used to recover target clones as described below.

### Primer design and PCR conditions

Adenylation and ketosynthase domains were amplified with degenerate primer pairs targeting previously described conserved regions^15^ with modifications in the degeneracy profile taking into account additional reference sequences reported in recent years. For enduracididine biosynthesis MppR, AHBA synthase, or Capreomycidine synthase domains, degenerate primers were designed to match two short conserved regions (18–23 bp) <1000 bp apart within their respective genes. The choice of priming site and degenerate nucleotide positions was guided by multiple sequence alignment of homologs found on NCBI. Multiple combinations of forward and reverse primers harboring different degeneracy levels, were tested at four annealing temperatures (55, 58, 61, and 64°C) in PCR reactions with soil DNA extracts. We used the highest degeneracy primer pair that produced an amplicon of the correct predicted size (as determined by agarose gel electrophoresis). The annealing temperature was further optimized using a temperature gradient and the lowest temperature yielding a distinct band was selected. To permit parallel sequencing of amplicons from multiple library subpools, we used 384 unique primer pairs that allow multiplexing 384 subpools as a single sample. Specifically, primers were composed of (i) an invariant landing pad for Illumina p5 or p7 sequence, (ii) a unique barcode (8 bp or 12 bp) that identifies a column (forward primer) or a row (reverse primer) in 384 sub-pools array, (iii) a spacer sequence required to phase amplicon sequence and increase bases diversity, (iv) a biosynthetic domain targeting degenerate sequence, as previously described. For each biosynthetic domain of interest, we designed a set composed of 24 barcoded forward/columns primers and 16 barcoded reverse/rows primers. Each primer set was arrayed in 384-well microplate to yield uniquely 384-barcoded primer pairs. Primers and barcode sequences are specified in Supplementary Data 2.

### Amplification of biosynthetic domains and targeted sequencing

As template for the targeted PCR amplification, metagenomic DNA cosmids were purified from each library subpool and arrayed in 384-well microplates, as described above. PCR amplification reactions were set in 384-well PCR plates using Viaflo (Integra) liquid handling platform. Each reaction contained 6 μl of FailSafe Buffer G x2 (Epicentre), 3.8 μl of water, 0.5 μl of each column/row barcoded primer (100 μM), 0.2 μl of rTaq polymerase (Bulldog Bio) and 1 μl of DNA from a specific subpool (100 ng/μl). PCR cycle conditions were set to: 95 °C 4 min, (95 °C 30 s, *T*_a_ °C 30 s, 72 °C 45 s) x 35 cycles, 72 °C 5 min. Specific annealing temperatures (*T*_a_) are detailed in Supplementary Table 2. PCR products were pooled as collections of 384 reactions from each plate, size-selected according to expected amplicon length, and used as a template for a second round of PCR to append plate specific sample indexes and sequencing adapters. Using suitable automation, the PCR amplification of targeted domains from thousands of subpools can be performed in 384 PCR plates and completed in a single day. The second PCR was set using 10 μl of FailSafe Buffer G x2 (Epicentre), 3.8 μl of water, 0.5 μl of Illumina universal forward (100 μM), 0.5 μl of indexed reverse primers (100 μM), 0.2 μl of Taq and 5 μl of purified amplicon product from the first round PCR (50 ng to 100 ng). PCR cycle conditions were set to: 95 °C for 5 min, (95 °C for 30 s, 70 °C for 30 s and 72 °C for 45 s) x 6 cycles, and finally, 72 °C for 5 min. Second round PCR amplicons were size-selected using Agencourt Ampure XP magnetic beads (Beckman Coulter) to remove excess sequencing adapter primers and quantified with an HS D1000 ScreenTape (Agilent 2200 TapeStation, Agilent Technologies). Purified second PCR products were mixed in an equal molar ratio to a final concentration of 4 nM and the resulting library was sequenced on a MiSeq instrument (MS-102-3003 or MS-103-1001, Illumina) according to standard amplicon sequencing workflow, with 10% phiX. A complete list of sequenced metagenomic libraries, number of subpools in each library, and sequencing yields are detailed in Supplementary Table 1.

### Domain clustering predictions from amplicon data

Following amplicon sequencing, raw fastq files were downloaded from BaseSpace (Illumina). Each sample file contains a multiplexed collection of amplicon reads from a specific PCR reaction plate (i.e., a collection of PCR amplicons from 384 library subpool reactions). Reads were demultiplexed to single subpool files based on the primer encoded barcodes using a custom Python script (https://github.com/brady-lab-rockefeller/paired-end-debarcoder). Forward reads (R1) were trimmed using VSEARCH^16^ (version 2.9.1) “-fastx_truncate” option with parameters “-stripleft primer_len_ -trunclen total_len_” to remove primer sequence and set a fixed read length (primer_len_ and total_len_ values for each amplified domain are detailed in Supplementary Table 2). Trimmed reads were de-replicated using VSEARCH “-fastx_uniques” option with “-size_out” flag for propagating de-replicated read counts. De-replicated reads from all library subpools were then clustered (95% sequence similarity cut-off) using VSEARCH “--cluster_size” option with “--id 0.95 --iddef 1 --sizein --sizeout --centroids --uc” parameters. The output clustering table was filtered based on the following criteria: (i) we remove sequences with read count smaller than the cut-off values detailed in Supplementary Table 2; (ii) we remove sequences with low read count (<5%) relative to the read count of the cluster centroid sequence within a cluster; (iii) we remove all clusters with reads originating from <3 distinct library wells, as these do not contain sufficient information to statistically infer co-occurrence patterns. When the same library was sequenced with multiple primer pairs (as shown in Fig. 4), the process was repeated for each dataset and all of the resulting amplicon clustering tables were merged prior to the joint analysis. The merged amplicon clustering table contains the list of domain variants (95% sequence identity clusters) identified in the sample and specifies the set of subpools in which each variant was detected in. To identify biosynthetic domains that originate from physically clustered metagenomic DNA, CONKAT-seq constructs the 2 × 2 contingency table for pairs of domain variants (specifying the number of subpools in which both domain variants, one of the two only, or none of them were identified) based on their subpool occurrences. Non-random co-occurrence of domain variants (i.e., domain pairs observed together more often than expected by chance under the null model of random, non-linked, and dispersal of domains across subpools) is statistically tested using one-sided Fisher’s exact test as implemented in the “fisher_exact” function in scipy.stats module. To reduce computation time for co-occurrence testing we limited our analysis to domain pairs co-occurring in at least three distinct library subpools. *P*-values were adjusted to control the false-discovery rate using a 2-stage Benjamini-Krieger-Yekutieli procedure as implemented in the “multipletests” function in statsmodels.stats module. Pairs of domains showing non-random association (adjusted *p*-value < 10^−6^) were considered to be physically linked, and hence predicted to belong to the same gene cluster. Based on a pairwise list of statistically significant links we constructed a graph representation domain networks where nodes represent cluster of biosynthetic domains and edges link domains that are predicted to be physically co-clustered. We note that biases in the sampling of domain variants are expected to arise due to differential amplification efficiencies and variability in sequencing depth between subpools. Such biases can contribute to a failure to detect co-occurring domains (false-negatives) but are less likely to generate false-positive associations. The analysis produced satisfactory results with the 2 types of library structures that we tested: 2,304 subpools of 5000 cosmids each (Arizona library) and 768 subpools of 25,000 cosmids each (New Mexico, Hawaii, Oregon libraries). While our empirical testing of CONKAT-seq results verified the vast majority of domain clustering predictions, we note that systematic errors during library preparation or in the assignment of amplicon reads to their subpools of origin (for example due to physical cross-contamination of library subpools or index-switching of barcoded reads) could result in the false clustering of unrelated domain pairs. In some cases we noted that sequencing errors and genetic diversity in domain sequences can lead to the emergence of highly similar variants within domain networks. To minimize the potential impact of downstream analysis bias due to a large number of closely similar sequences, we enabled CONKAT-seq “merge_similar_nodes” mode which merges similar domain variants (>90% identify threshold) within each domain network. All scripts required for CONKAT-seq analysis are available online (https://github.com/brady-lab-rockefeller/conkat_seq).

### Library subpools sequencing using long reads technology

Arizona library subpools 2185 and 2248 were inoculated from glycerol stocks in 3 mL of LB supplemented with 12.5 μg/mL chloramphenicol and cultured overnight. Cosmid DNA was isolated by miniprep (QIAprep, Qiagen), sheared to 6–20 kb with g-Tube (Covaris), and prepared with SMRTbell Express Template Prep Kit (Pacific Biosciences). The libraries were sequenced using SMRTCell 1 M v2 Trays on a PacBio Sequel System (Pacific Biosciences) to generate ≈12 Gbp per subpool. Data were processed using minimap2 (https://github.com/lh3/minimap2), SAMtools, Jvarkit (http://lindenb.github.io/jvarkit/SamExtractClip) and Flye^17^. Briefly, subreads were aligned on 2000bp of pWEB_TNC vector sequence and the *E. coli* chromosome using minimap2 with default parameters. Non-aligned subreads were kept in full while aligned subreads were processed with Jvarkit’s SamExtractClip with parameter --minsize 1000 to recover only their non-aligned regions (i.e. metagenomic inserts regions clipped by minimap2). Following vector and residual *E. coli* DNA filtering, the subreads were assembled using Flye with parameter “--genome-size 200 m”. Resulting contigs longer than 25 kb harboring at least one vector edge were used in downstream analysis (predicted domain detection and abundance estimation). Analysis script is available online (https://github.com/brady-lab-rockefeller/BGCs_in_rare_metagenomic_DNA/).

### Recovery of BGC encoding clones from metagenomic library subpools

For each predicted domain network (i.e., a collection of biosynthetic domains predicted to be chromosomally clustered in the metagenome) CONKAT-seq specifies a set of library subpools from which the BGC encoding metagenomic DNA can be physically recovered. Specific primer pairs were designed for each target clone based on the amplicon sequence of one of the biosynthetic domains in the domain network. Serial dilution PCR was then used to isolate and recover the BGC encoding target clone from the relevant library subpool according to the following procedure. The library subpool containing the target clone was inoculated from frozen glycerol stock in LB media supplemented with chloramphenicol (12.5 μg/mL), incubated overnight to saturation, and diluted to a concentration of 4000 CFU/mL. Next, 384 microplate wells were inoculated with 10 μL (40 CFU) of the resulting dilution per well and incubated overnight to saturation. The diluted cultures, containing 40 distinct library clones each, were screened using real-time PCR to identify wells containing the target clone. Target positive wells were plated on solid medium and the target clone was identified by colony PCR. Positive colonies were inoculated in 8 mL of LB media supplemented with chloramphenicol and incubated overnight. The isolation of a BGC containing target clone from frozen glycerol stock of the corresponding subpool, to a monoculture of the *E. coli* clone harboring the target cosmid can be typically accomplished within 5 days. If multiple clones of interest have been identified, their recovery can be conducted in parallel. The cosmid harboring the targeted metagenomic DNA was purified from the cultures (QIAprep, Qiagen) and sequenced. In brief, metagenomic DNA cosmids isolated from the library were treated with RNase and quantified using the Qubit dsDNA HS Assay System (Q32854, ThermoFisher Scientific). Multiplexed sequencing libraries were prepared using a Nextera XT DNA Sample Preparation Kit (FC-131-1024) with Nextera XT Index kit (FC-131-1001) based on protocols provided by the manufacturer (Illumina) in batches of 96 cosmids per sequencing run. Each library was pooled as collection of 96 samples and the quality of final library pool was verified using HS D1000 ScreenTape (TapeStation 2200, Agilent Technologies). The resulting library was sequenced using MiSeq Reagent Nano Kit v2 (MS-103-1001, Illumina) on a MiSeq sequencer (Illumina). Reads were assembled into contigs using Newbler 2.6 (Roche). Fully assembled cosmids were processed using antiSMASH 4.1 with default parameters^18^.

### Comparison of domain network predictions with contigs

Metagenomic BGC sequences that correspond to specific predicted domain networks, obtained either from long-read assembly contigs or from fully sequenced recovered library clones, were annotated with antiSMASH 4.1. For each predicted network, domain amplicon sequences (nodes) that originate from the subpool from which the BGC was recovered were mapped to the corresponding BGC sequence using VSEARCH “--usearch_global” option with a 93% sequence identity match threshold. Network domains that match the BGC sequence were considered as “validated clustering” predictions while network domains that were not present in the BGC sequence were considered as “false clustering” predictions. A detailed description of the validated domain networks appearing in Fig. 2b can be found in Supplementary Data 1.

### Comparison of domain networks to references and similarity score

For each soil sample, sequenced amplicons forming networks of 3 or more domains were translated into protein sequences and compared to the protein sequence of BGCs in databases (MIBiG and AntismashDB) or to predicted domain networks from other soil samples using blastp with parameters “-evalue 1e-20 -qcov_hsp_perc 80 -max_target_seqs 200000000”. If at least 50% of the domains in a network matched independent positions on proteins from a BGC in a database or independent domains in another domain network, a similarity score was calculated for the pair. The similarity score is defined as the median pairwise identity between domains and reference BGC proteins, taking into account non-matching domains and the best combination of matching domains. The script used to calculate similarity scores used in Figs. 2a and 3a, and Supplementary Table 3 is available online (https://github.com/brady-lab-rockefeller/BGCs_in_rare_metagenomic_DNA/). Figure 3a was generated using chord2.js, a d3.js plugin developed by G. Gherdovich (https://bitbucket.org/gghh/chord2/).

### Untargeted sequencing of metagenomic library and abundance estimation

For shallow depth sequencing of the metagenomic library constructed from the Arizona soil sample, 10 μl of saturated culture were pooled from each library subpool (following transfection with cosmids containing metagenomic DNA). Cosmid DNA was purified from 8 ml of the pooled metagenomic library (containing all 10^7^ clones), and a single sequencing library was prepared as described for the clonal cosmids. The library was sequenced using an Illumina HiSeq 2500 platform to generate 296 million single reads of 100 bps. Data processing was made with a custom Python script available online (https://github.com/brady-lab-rockefeller/BGCs_in_rare_metagenomic_DNA/) which relied on BBtools (Bushnell B. sourceforge.net/projects/bbmap/), SAMtools (https://sourceforge.net/projects/samtools/) and bedtools (https://bedtools.readthedocs.io/). In brief, duplicate reads were removed using BBtools’s clumpify.sh with parameters “dedupe subs = 2” and reads originating from pWEB_TNC vector or residual *E. coli* chromosomal DNA were detected and filtered out by aligning them using bbmap.sh with parameters “maxindel = 80 minid = 0.95”. 153 millions reads were retained for downstream analysis (15.5Gbp). Reads were aligned on all contigs of interest (50 library clones encoding short or partial BGCs, 8 full BGCs obtained from multiple overlapping cosmids, 3760 untargeted metagenomic inserts obtained from long reads assembly) using bbmap.sh with parameters “maxindel = 80 minid = 0.95 ambig = all secondary = t saa = f ssao = t sssr = 0.95”. For each contig of interest, aligned reads were extracted with SAMtools, and coverage for each base pair was obtained using bedtools’ genomeCoverageBed in order to calculate a mean coverage per base pair (depth of coverage). Based on the depth of coverage obtained by sequencing 15.5 Gbp, the abundance of the genomes of origin of each contig was extrapolated and expressed as the sequencing output required to obtain a depth of coverage of 20 (required output = 15.5/Obtained coverage * 20). The frequency of the genome of origin of a BGC in the library based on the obtained coverage was estimated assuming a 10 Mbp genome (frequency = 1/((15.5/0.01)/Obtained coverage).

### Validation of the classification of BGCs into families

Recovered BGC sequences were annotated with antiSMASH 4.1, and BiG-SCAPE (version 20181005 https://git.wageningenur.nl/medema-group/BiG-SCAPE/) was used with a cut-off parameter of 0.5 to compare each recovered BGC to each other and to public datasets of sequenced BGCs (antismashDB v2, MIBiG v1.4). Recovered BGCs that did not form any link to reference BGCs with a raw distance below 0.5 were considered novel.

### TAR Assembly of complete BGCs from multiple cosmids

For the assembly of BGCs from multiple library clones we used transformation-associated recombination (TAR) in yeast, as previously described^19^. Briefly, overlapping cosmids containing the full biosynthetic pathway were digested and linearized with DraI. Two homology arms to the terminal overlapping cosmid clones (≈500 bp each) were cloned into a shuttle capture vector (pTARa). The capture vector was linearized with PmeI and gel-purified. The digested BGC containing cosmids were co-transformed together with the linearized pTARa vector into 200 μL of Saccharomyces cerevisiae CRY1-2 spheroplasts prepared according to published methods^20^. Transformed spheroplasts were overlaid onto synthetic complete top agar plates depleted of lysine and incubated at 30 °C until colonies appeared. Yeast colonies were screened using primer pairs that target regions from each of the assembled cosmids and pTARa-assembled DNA was isolated from PCR-positive yeast clones. The assembly product was transformed into *E. coli* ET12567/pUZ8002 cells and conjugated into *Streptomyces spp*. for heterologous expression.

### Heterologous expression of TAR assembled BGCs

TAR assembled BGCs and an empty pTARa vector control were separately integrated into the chromosome of Streptomyces albus J1074. Spore suspensions of these recombinant strains were used to seed starter cultures in 5 mL trypticase soy broth (Oxoid). These cultures were grown for 48 h (30 °C/200 rpm) and 0.4 mL of the resulting confluent culture was used to inoculate 50 mL of R5a production medium containing: 100 g/L sucrose, 10 g/L d-glucose, 5 g/L yeast extract, 10.12 g/L MgCl_2_·6H_2_O, 0.25 g/L K_2_SO_4_, 0.1 g/L casamino acids, 21 g/L MOPS, 2 g/L NaOH, 5.88 mg/L CaCl_2_, 80 μg/L ZnCl_2_, 400 μg/L FeCl_3_·6H_2_O, 20 μg/L MnCl_2_, 20 μg/L CuCl_2_, 20 μg/L Na_2_B_4_O_7_·10H_2_O, 20 μg/L (NH_4_)_6_Mo_7_O_24_·4H_2_O, pH = 6.85. Cultures were fermented in 125 mL baffled flasks (30 °C, 220 rpm) for 14 days.

### Scale-up fermentation, extraction and isolation of omnipeptin

Spore suspensions of *S. albus* J1074 conjugated with the omnipeptin biosynthetic gene cluster were inoculated into 50 mL of trypticase soy broth (TSB) and shaken (200 rpm) for 48 h at 30 °C. 6 mL of seed culture was transferred into an 2.8 L baffled Fernbach flasks containing 600 mL of R5A broth (100 g/L sucrose, 10 g/L d-glucose, 5 g/L yeast extract, 10.12 g/L MgCl_2_·6H_2_O, 0.25 g/L K_2_SO_4_, 0.1 g/L casamino acids, 21 g/L MOPS, 2 g/L NaOH, 5.88 mg/L CaCl_2_, 80 μg/L ZnCl_2_, 400 μg/L FeCl_3_·6H_2_O, 20 μg/L MnCl_2_, 20 μg/L CuCl_2_, 20 μg/L Na_2_B_4_O_7_·10H_2_O, 20 μg/L (NH_4_)_6_Mo_7_O_24_·4H_2_O, pH = 6.85) and 20 g of autoclaved HP20 resin. After 10 days of shaking (200 rpm) at 30 °C, the resin was collected with cheesecloth from 8 Fernbach flasks and dried at 30 °C for 24 h. The dried resin was packed into a column, washed with 2 L of H_2_O, and eluted with 2 L of methanol. The methanolic elution was concentrated in vacuo at 30 °C and adsorbed onto C18 reversed phase silica gel. The crude extract was initially partitioned by medium-pressure liquid chromatography (50 g Gold HP C18 column, 90% H_2_O/MeOH isocratic elution for 1 min, followed by a gradient elution from 90% to 10% H_2_O/MeOH for 18 min, 40 mL/min). Fractions were analyzed by UPLC-DAD-MS (Acquity UPLC BEH C18, 2.1 × 50 mm, 1.7 μm, 130 Å, 0.6 mL/min gradient elution from 95% H_2_O/MeCN to MeCN over 10 min, with 0.1% formic acid; positive and negative ionization modes) and those containing peaks for omnipeptin were combined. The combined fractions (123.8 mg) were further subjected to Sephadex LH-20 chromatography and eluted with methanol. The Sephadex LH-20 column fraction (76.8 mg) containing omnipeptin was subjected to HPLC chromatography (XBridge Prep C18, 10 × 150 mm, 5 μm, 130 Å, 3.5 mL/min gradient elution from 75% to 55% H_2_O/MeCN over 50 min, with 0.1% formic acid) to afford the pure form of omnipeptin (9.4 mg, *t*_R_ = 34.7 min).

### Reporting summary

Further information on research design is available in the Nature Research Reporting Summary linked to this article.

## Supplementary information

Supplementary Information

Description of Additional Supplementary Files

Supplementary Data 1

Supplementary Data 2

Reporting Summary

Source Data

## Data Availability

All sequences of metagenomic inserts harboring BGCs reported in this work are available from Genbank (Accession numbers MN161598 to MN161666) and from github (https://github.com/brady-lab-rockefeller/BGCs_in_rare_metagenomic_DNA). Demultiplexed reads of adenylation domains and domain networks from the Arizona library are available from github (https://github.com/brady-lab-rockefeller/BGCs_in_rare_metagenomic_DNA). The remaining data that support the findings of this study are available from the corresponding author upon request.

## Code availability

All scripts developed for this work are publicly available from github (https://github.com/brady-lab-rockefeller/conkat_seq and https://github.com/brady-lab-rockefeller/BGCs_in_rare_metagenomic_DNA). Analysis was performed using Python 2.7.15 (Anaconda) with the following module dependencies: numpy (1.14.3), pandas (0.23.0), networkx (2.1), BioPython (1.68), scipy (1.1.0) and statsmodels (0.9.0). Amplicon sequences were demultiplexed using a Python script available at https://github.com/brady-lab-rockefeller/paired-end-debarcoder. Primer removal, amplicon length trimming and clustering were performed using VSEARCH (version 2.9.1). Alignment of reads to reference sequences was performed using BBmap (version 38.22), SAMtools (version 1.9), and bedtools (version 2.27.1). Biosynthetic domains were compared to each other and to reference sequences using blastp (version 2.6.0+). Statistical analysis of amplicon co-occurrences was performed using one-sided Fisher’s exact test as implemented in the “fisher_exact” function in scipy.stats module. P-values were adjusted to control the false-discovery rate using a 2-stage Benjamini-Krieger-Yekutieli procedure as implemented in the “multipletests” function in statsmodels.stats module. Detection and annotation of biosynthetic gene clusters was performed using antiSMASH (version 4.1). Similarity metric scores between biosynthetic gene clusters was calculated using BiG-SCAPE (version 20181005). Domain networks were visualized with Cytoscape (version 3.7.1).
