# Supplementary material for: Uncovering the biosynthetic potential of rare metagenomic DNA using co-occurrence network analysis of targeted sequences
Source: Nat Commun. 2019 Aug 26;10:3848. doi: 10.1038/s41467-019-11658-z (PMC6710260; doi:10.1038/s41467-019-11658-z)
Supplement: Supplementary file 1 — Supplementary Information [file 41467_2019_11658_MOESM1_ESM.pdf]

Supplemental Information for:

Title: Uncovering the biosynthetic potential of rare metagenomic DNA using co-occurrence network analysis of targeted sequences

Authors: Vincent Libis[1], Niv Antonovsky[1], Mengyin Zhang, Zhuo Shang, Daniel Montiel, Jeffrey Maniko, Melinda A. Ternei, Paula Y. Calle, Christophe Lemetre, Jeremy G. Owen and Sean F. Brady*

Author affiliation:
Laboratory of Genetically Encoded Small Molecules, The Rockefeller University, 1230 York Avenue, New York, NY 10065.

[1] These authors contributed equally to this work.

***Corresponding Author:** Sean F. Brady
Contact: Laboratory of Genetically Encoded Small Molecules
The Rockefeller University
1230 York Avenue
New York, NY 10065
Phone: 212-327-8280
Fax: 212-327-8281
Email: sbrady@rockefeller.edu

Supplementary Table 1: Soil samples and metagenomic libraries information

Library name	subpools	clones per subpool	library clones	avg. insert size [kbp]	total library size [Gbp]	amplicon sequencing [M reads]				
						adenylation (NRPS)	ketosynthase (PKS)	enduracididine	AHBA	capreomycidine
Arizona	2304	5000	1E+07	40	400	24	11*	0.2	0.1	15
Oregon	768	25000	2E+07	40	800	11	10	0.2	N/A	N/A
New Mexico	768	25000	2E+07	40	800	12	11	N/A	0.05	N/A
Hawaii	768	25000	2E+07	40	800	11	N/A	N/A	N/A	N/A

*Due to low sequencing coverage of one 384 plate, only 1920 subpools were processed for Arizona ketosynthase domains

Supplementary Table 2: Primers table

Target domain	Annealing T°	Forward binding sequence	Reverse binding sequence	$Primer_{len}$ / $Total_{len}$
AD1 (Arizona)	65	5′-GCSTACSYSATSTACACSTCSGG	5′-SASGTCVCCSGTSCGGTA	23 / 210
AD2 (Oregon, Hawaii, New Mexico)	56.3	5′-SATBTAYACSTCVGGHWCSAC	5′-CCANRTCNCCBGTSYKGTACA	21 / 210
KS (Arizona, Oregon, Hawaii, New Mexico)	56.3	5′-GCNATGGAYCCNCARCARMGNVT	5′-GTNCNNGTNCCRTGNSCYTCNAC	23 / 210
END. (Arizona, Oregon)	64.5	5′-CCNCCRCCSTGGCACTTCKCSG	5′-CSAACTGCTTGGGCATSCCCTG	22 / 110
AHBA (Arizona, New Mexico)	61	5′-CAGAACGGCAAGCTGATGACSG	5′-GGAMCATSGCCATGTAGYKSG	22 / 110
CAP. (Arizona)	55	5′-SGACRTSWSCWSSWSCGGYGT	5′-CRSTNGGRTTRTGSGGRAAGT	21 / 210

Supplementary Table 3: BGCs similarity analysis of 60 metagenomic clones

Recovered BGC #	Highest median identity to a reference BGC (%)	BiG-SCAPE Raw distance	Closest relative (BiG-SCAPE)	Source organism
**29:Genbank MN161611**	95.6	0.36	GCF_001886595_NZ_CP018074.1.cluster027	*Streptomyces venezuelae* NRRL B-65442
**9:Genbank MN161603**	95.6	0.36	GCF_000023245_NC_013093.1.cluster022	*Actinosynnema mirum* DSM 43827
**28:Genbank MN161610**	83.5	0.43	GCF_001302585_NZ_CP012752.1.cluster008	*Kibdelosporangium phytohabitans*
**38:Genbank MN161613**	79.7	0.43	GCF_001579845_NZ_CP007440.1.cluster002	*Rhodoplanes sp.* Z2-YC6860
**25:Genbank MN161608**	89.8	0.43	GCF_001443625_NZ_CP013129.1.cluster026	*Streptomyces venezuelae*
**73:Genbank MN161622**	89.7	0.44	GCF_002796545_NZ_CP024894.1.cluster009	*Amycolatopsis sp.* AA4
**355:Genbank MN161648**	94.2	0.45	GCF_000282715_NC_018266.1.cluster017	*Amycolatopsis mediterranei* S699
**46:Genbank MN161620**	89.8	0.45	GCF_001302585_NZ_CP012752.1.cluster017	*Kibdelosporangium phytohabitans*
**900:Genbank MN161659**	88.4	0.47	GCF_000282715_NC_018266.1.cluster017	*Amycolatopsis mediterranei* S699
**16:Genbank MN161605**	92.7	0.48	GCF_000739085_NZ_CP009110.1.cluster003	*Amycolatopsis methanolica* 239
**41:Genbank MN161615**	97.1	0.49	GCF_001650215_NZ_CP015726.1.cluster030	*Streptomyces sp.* RTd22
**40:Genbank MN161614**	85.5	>0.5	NA	NA
**47:Genbank MN161621**	84	>0.5	NA	NA
**96:Genbank MN161625**	76.8	>0.5	NA	NA
**683:Genbank MN161657**	75.7	>0.5	NA	NA
**917:Genbank MN161660**	75.3	>0.5	NA	NA
**All remaining BGCs (44)**	<75	>0.5	NA	NA

Supplementary Table 4: Omnipeptin biosynthetic gene analysis

Gene	Proposed function	NCBI similarity	Species	%ID
omn1		flavin reductase	*Kibdelosporangium sp. MJ126-NF4*	71
omn2		cation/H(+) antiporter	*Amycolatopsis antarctica*	45
omn3	Halogenation of tryptophan	tryptophan 7-halogenase	*Streptomyces sp. NRRL S-146*	76
omn4	fatty acyl-AMP ligase	fatty acyl-AMP ligase	*Streptomyces formicae*	65
omn5	Attachment of acyl chain	acyl carrier protein	*Streptomyces caeruleatus*	49
omn6	NRPS	non-ribosomal peptide synthetase	*Sciscionella sp. SE31*	53
omn7	NRPS	non-ribosomal peptide synthetase	*Actinoplanes friuliensis*	50
omn8	NRPS	non-ribosomal peptide synthetase	*Streptomyces sp. LUP47B*	57
omn9		protein mbtH	*Saccharomonospora viridis*	68
omn10	Deacylation	Cyclic lipopeptide acylase	*Streptomyces canus*	55
omn11		alpha/beta hydrolase	*Streptomyces sp. M1013*	67
omn12		hypothetical protein	*Streptomyces sp. LUP47B*	61
omn13		hypothetical protein	*Sciscionella sp. SE31*	38
omn14	Transport	ABC transporter permease	*Herbihabitans rhizosphaerae*	66
omn15	Transport	daunorubicin resistance protein DrrA family ABC transporter ATP-binding protein	*Saccharothrix espanaensis*	65
omn16		glutamate synthase	*Amycolatopsis mediterranei*	70
omn17		asparagine ligase	*Saccharothrix syringae*	75
omn18		methylaspartate mutase E	*Streptomyces pratensis ATCC 33331*	64
omn19		methylaspartate mutase S	*Streptomyces erythrochromogenes*	59
omn20	Regulation	ArsR family transcriptional regulator	*Actinoplanes utahensis*	43
omn21		hypothetical protein	*Streptomyces canus*	62
omn22	Transport	ABC transporter permease	*Streptomyces sp. LUP47B*	59
omn23	Transport	ABC transporter ATP-binding protein	*Streptomyces sp. LUP47B*	76
omn24	Regulation	signal transduction histidine kinase	*Actinokineospora cianjurensis*	60
omn25	Regulation	response regulator transcription factor	*Actinokineospora cianjurensis*	80
omn26	Hydroxylation of tyrosine	cytochrome P450	*Streptomyces sp. NRRL B-24051*	64
omn27	Hydroxylation of proline	proline hydroxylase	*Streptomyces malaysiensis*	53
omn28		phytanoyl-CoA dioxygenase	*Streptomyces sp. H23*	66
omn29		alcohol dehydrogenase	*Streptomyces sp. LUP47B*	73
omn30		myo-inositol-1-phosphate synthase	*Kibdelosporangium aridum*	64
omn31		4-hydroxybenzoate polyprenyltransferase	*Actinocrispum wychmicini*	57
omn32		sugar phosphate isomerase/epimerase	*Actinosynnema sp. ALI-1.44*	70
omn33		TatD family hydrolase	*Amycolatopsis nigrescens*	79
omn34		xylose isomerase	*Kibdelosporangium aridum*	67
omn35		alkaline phosphatase family protein	*Prauserella shujinwangii*	72
omn36	Regulation	AfsR/SARP family transcriptional regulator	*Kibdelosporangium phytohabitans*	60
omn37	Regulation	TetR family transcriptional regulator	*Actinophytocola oryzae*	47
omn38	Regulation	TetR/AcrR family transcriptional regulator	*Kibdelosporangium aridum*	66
omn39		gamma-butyrolactone biosynthesis protein	*Streptomyces sp. RP5T*	49
omn40		NAD-dependent epimerase/dehydratase family protein	*Herbihabitans rhizosphaerae*	56

Supplementary Table 5. ^{1}H and ^{13}C NMR (600 MHz, DMSO-d_6) data for omnipeptin (**1**)

**Residue**	**Pos.**	**δ_C**	**δ_H, mult. (J in Hz)***	**Residue**	**Pos.**	**δ_C**	**δ_H, mult. (J in Hz)***
isoAsp-1	1	170.9, C	−		5-CO$\underline{NH_2}$	−	6.90, brs; 7.32
	2	50.9, CH	3.57		2-NH	−	7.87, br s
	3	36.4, CH_2	2.19, 2.75	Cl-Trp-8	1	170.7, C	−
	4-CONH	169.1, C	−		2	53.4, CH	4.55
hyTyr-2	1	170.4, C	−		3	27.7, CH_2	2.85, 3.13
	2	59.9, CH	4.28, d (7.4)		4	110.3, C	−
	3	72.4, CH	4.66, d (7.4)		4a	126.0, C	−
	4	132.1, C	−		5	119.8, C	7.58, d (8.4)
	5/9	128.0, CH	7.22		6	118.6, CH	6.97, d (8.4)
	6/8	114.5, CH	6.65, d (7.9)		7	125.6, C	−
	7	156.7, C	−		8	110.8, CH	7.34, s
	2-NH	−	8.52, br s		8a	136.5, C	−
Ser-3	1	169.1, C	−		9	125.0, CH	7.19
	2	52.0, CH	4.58		2-NH	−	7.64
	3	64.0, CH_2	4.34, d (9.9); 4.40		9-NH	−	11.0, s
	2-NH	−	8.23, d (6.6)	Val-9	1	170.2, C	−
Glu-4	1	171.5, C	−		2	57.6, CH	4.05
	2	52.5, CH	4.47		3	30.3, CH	1.66, m
	3	27.1, CH_2	1.81, m; 2.02, m		4	18.8, CH_3	0.39, d (6.5)
	4	30.6, CH_2	2.14, 2.23		5	17.8, CH_3	0.47, d (5.9)
	5-COOH	174.5, C	−		NH	−	7.70, br s
	2-NH	−	8.13, br s	Phe-10	1	170.6, C	−
Thr-5	1	170.4, C	−		2	51.1, CH	4.74, br s
	2	57.9, CH	4.43		3	36.5, CH_2	2.76; 2.98, d (11.8)
	3	66.7, CH	4.05		4	137.5, C	−
	4	19.6, CH_3	1.01, d (6.1)		5/9	129.4, CH	7.31, d (7.7)
	2-NH	−	7.67		6/8	128.0, CH	7.23
Ser-6	1	170.8, C	−		7	126.3, CH	7.16
	2	54.7, CH	4.46		NH	−	8.37
	3	61.5, CH_2	3.51, br s; 3.69, dd (10.1, 5.7)	MehyPro-11	1	169.5, C	−
	2-NH	−	8.36		2	62.3, CH	4.37, d (7.8)
MeAsn-7	1	169.6, C	−		3	74.9, CH	4.21, br s
	2	55.2, CH	4.47		4	37.5, CH	2.21
	3	40.1, CH	2.68, m		5	50.6, CH_2	3.20, 3.81, br s
	4	11.8, CH_3	0.66, br s		6	14.3, CH_3	1.04, d (6.1)
	5-$\underline{CO}NH_2$	175.0, C	−				

* The assignments of overlapping ^{1}H NMR signals were supported by HSQC, HMBC, TOCSY and HSQC-TOCSY. The peak multiplicity and coupling constants are presented for non-overlapping signals only.

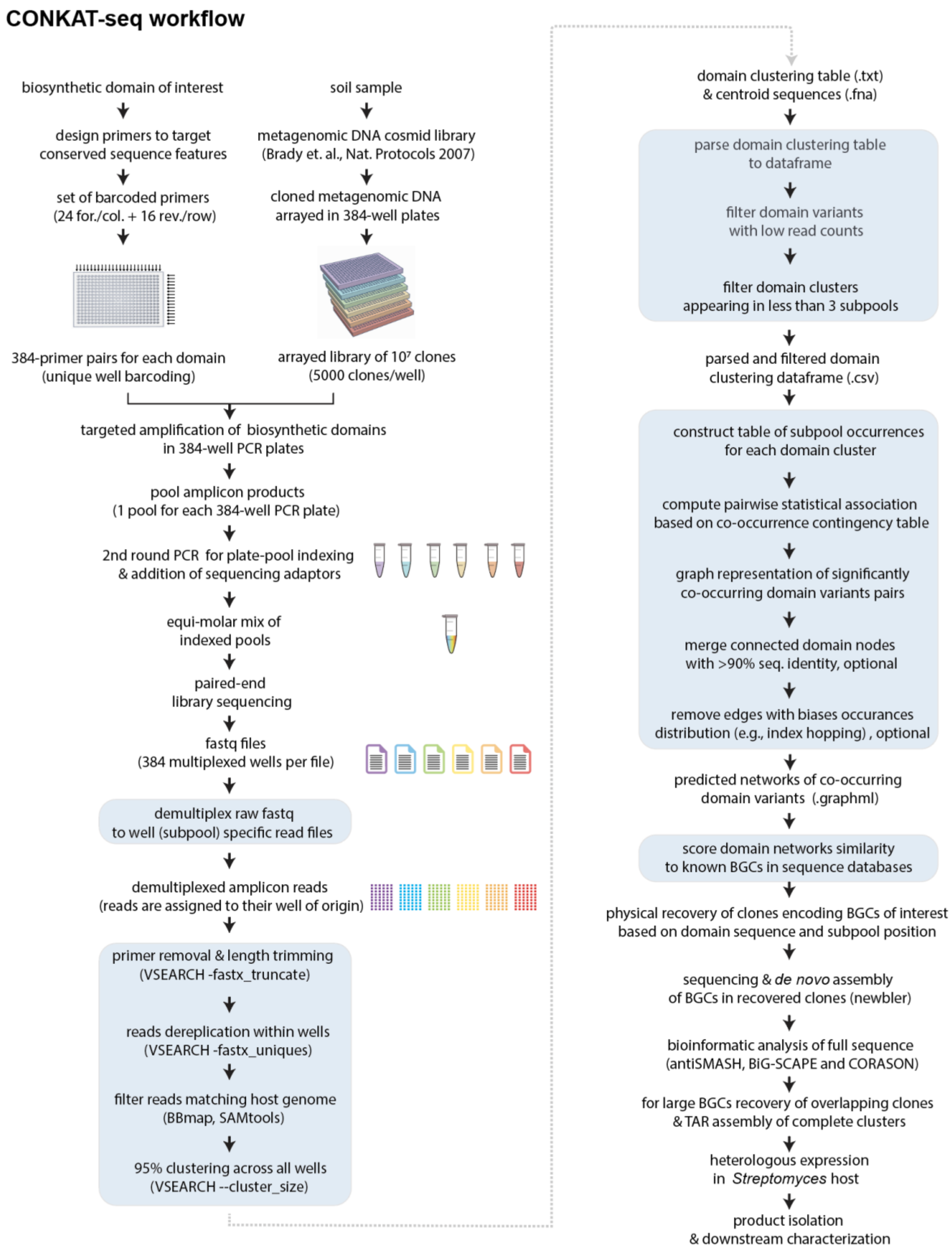

Supplementary Fig. 1 - CONKAT-seq workflow. CONKAT is based on the statistical analysis of amplicon co-occurrence in a partitioned library of large insert metagenomic clones. High molecular weight DNA from soil samples are extracted and cloned to construct large insert metagenomic library which preserves the linkage between co-clustered genes. Library clones are randomly partitioned into hundreds of wells (subpools), and DNA sequence encoding for biosynthetic domains of interest are amplified using barcoded primers. Amplicon de-barcoding identifies the positioning of each biosynthetic domain within the array of subpools and enables the statistical analysis of domain co-occurrence. Domains encoded within a BGC are expected to show high level of co-occurrence across subpools due to their physical linkage. Significantly associated domains are grouped into networks, that can be guide the recovery of novel BGCs based on their similarity scores to known BGCs. The domain variant sequences and the subpool localization information that are associated with networks of interest can guide the physical recovery of metagenomic clones encoding for novel BGCs from the library using serial dilution PCR strategy with domain variant specific primers. Isolated metagenomic clones can be sequenced and assembled to obtain the full BGC sequence encoded within the clone, or heterologously expressed in a suitable host to attempt and obtain the molecular product. Scripts for domain clustering prediction from amplicon data (highlighted in blue) are available at https://github.com/brady-lab-rockefeller/conkat_seq.

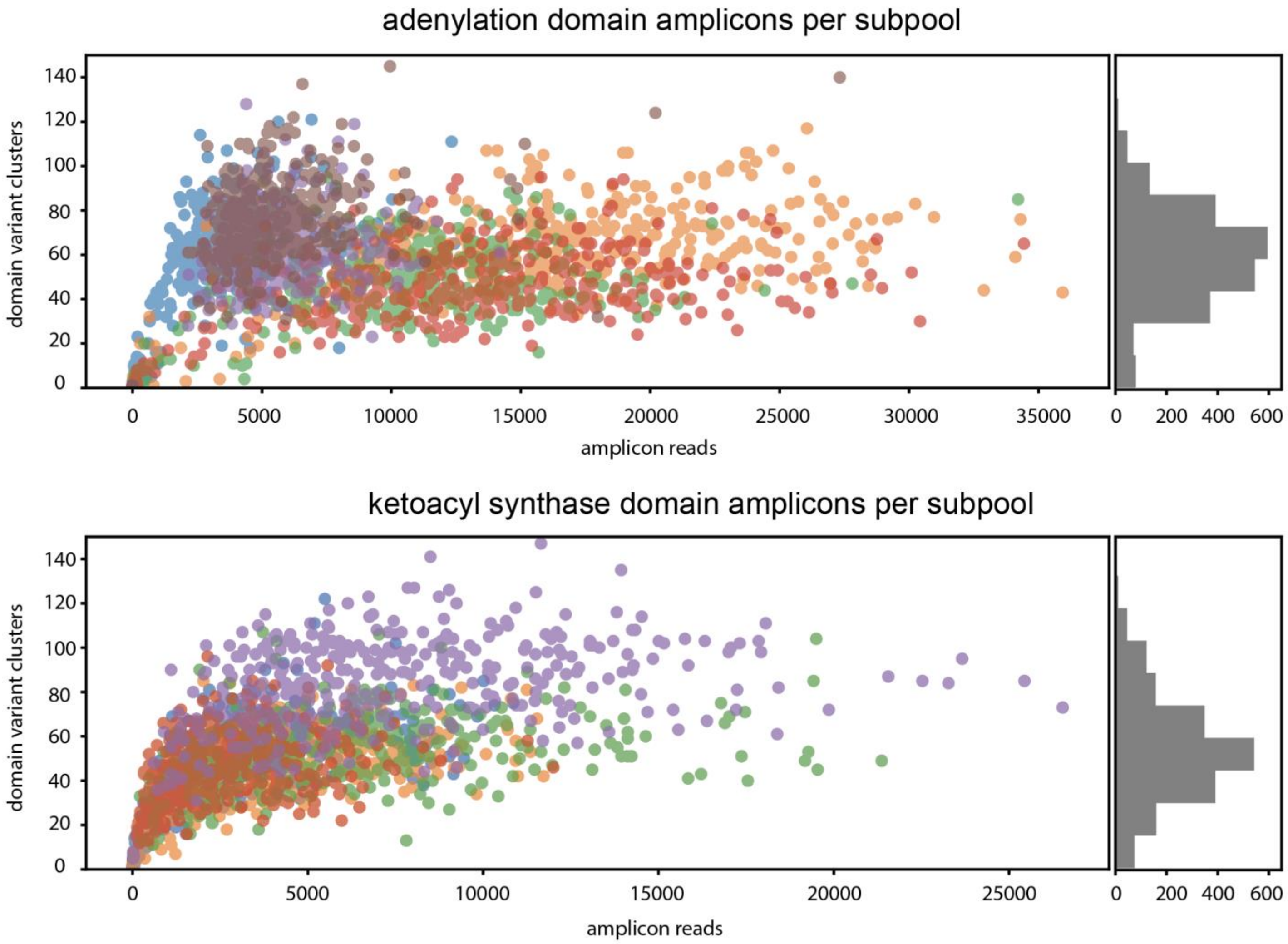

Supplementary Fig. 2 - Adenylation and ketoacyl-synthase domain variants in the Arizona library subpools. Scatter plot of amplicons reads and domain variant clusters (95% identity threshold) in subpools of the Arizona metagenomic library. Each subpool (containing ~5000 metagenomic clones) was PCR amplified using adenylation and ketoacyl-synthase subpool-barcoed domain primers. Approximately 3000 reads per subpool were required to saturate the diversity of domain variants amplified by these primers in each subpool. On average, we identified 60 adenylation domain variants and 55 ketoacyl-synthase domain variants per subpool and a total of 10^5 unique domain variants across the library. Colors represent 384-subpools PCR reaction plates that have been pooled and sequenced as an indexed sample.

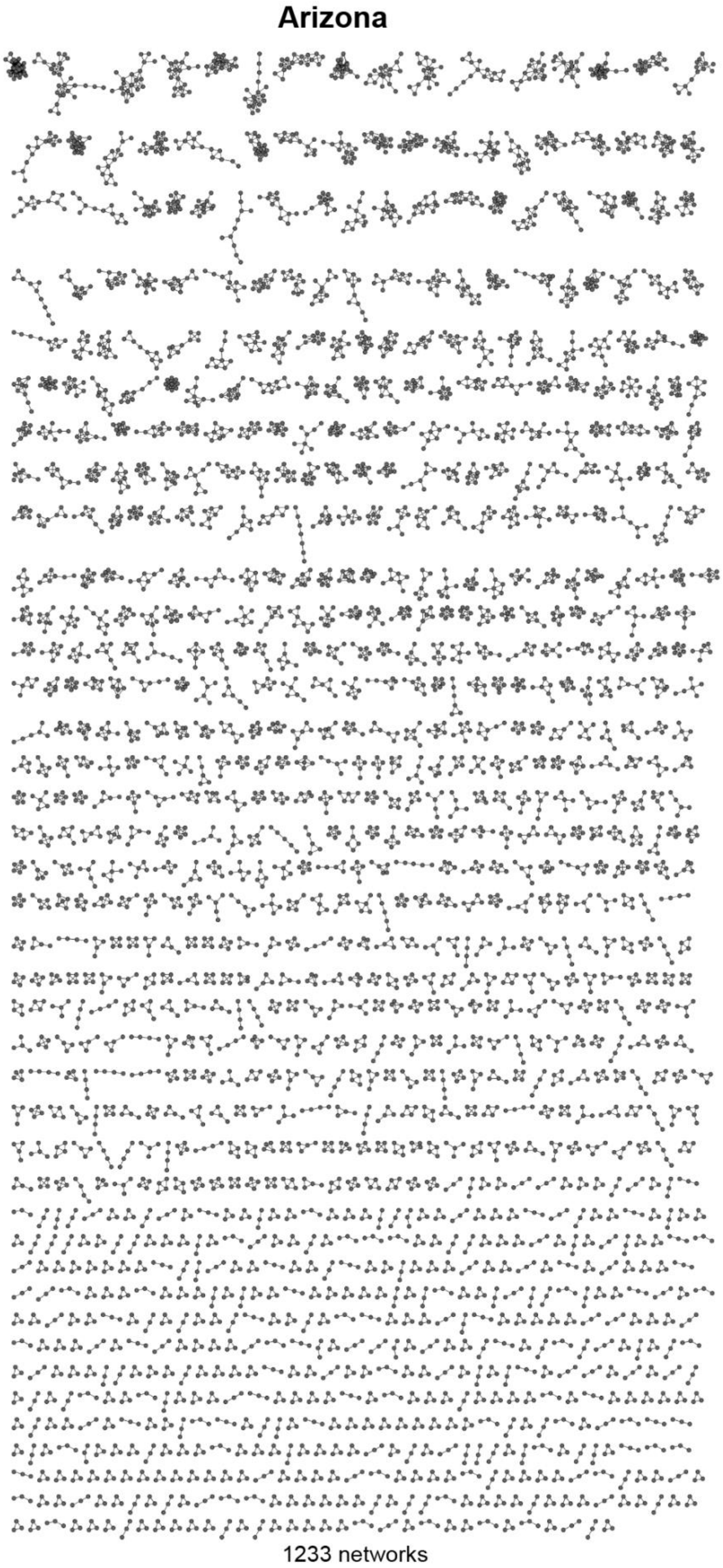

Supplementary Fig. 3 - Predicted domain networks (Arizona)

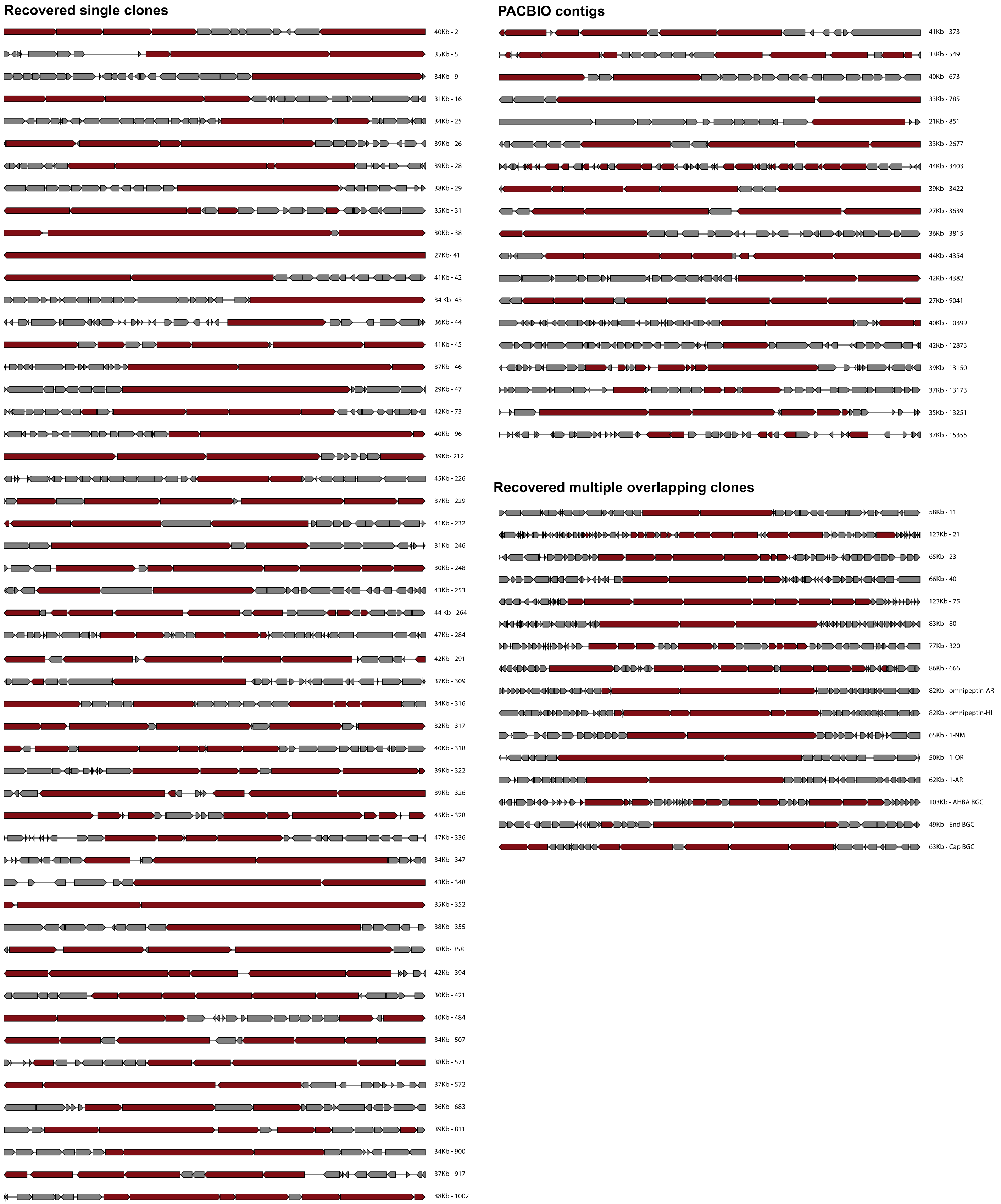

Supplementary Fig. 4 - NRPS and PKS genes locations in metagenomic insert sequences. NRPS and PKS genes are depicted in red. Recovered clones have been sequenced individually with short-read technology (Illumina) while PacBio contigs have been obtained from bulk sequencing of 2 subpools of ~5000 clones each with long-read technology.

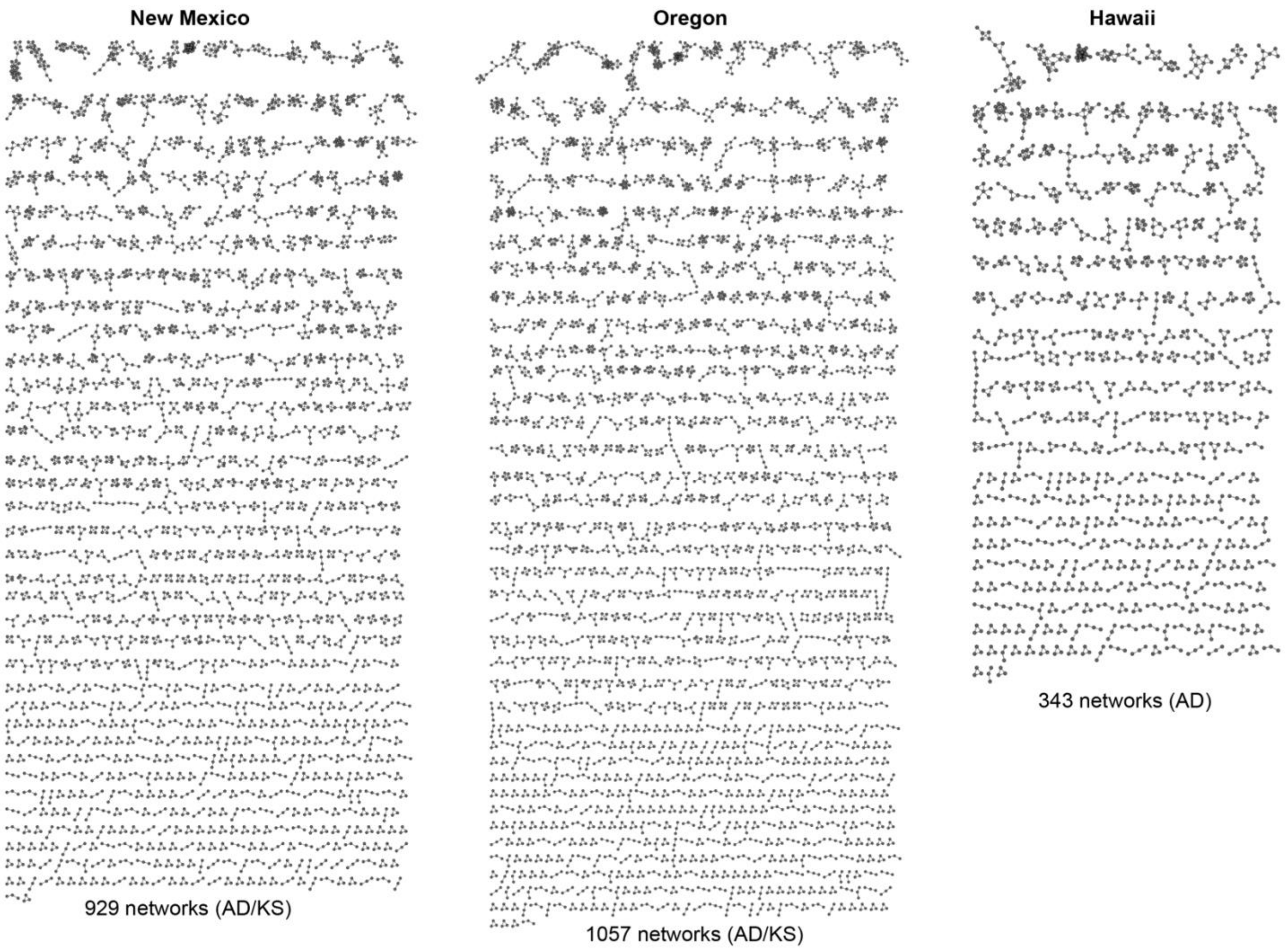

Supplementary Fig. 5 - Predicted domain networks (Oregon, New Mexico, Hawaii)

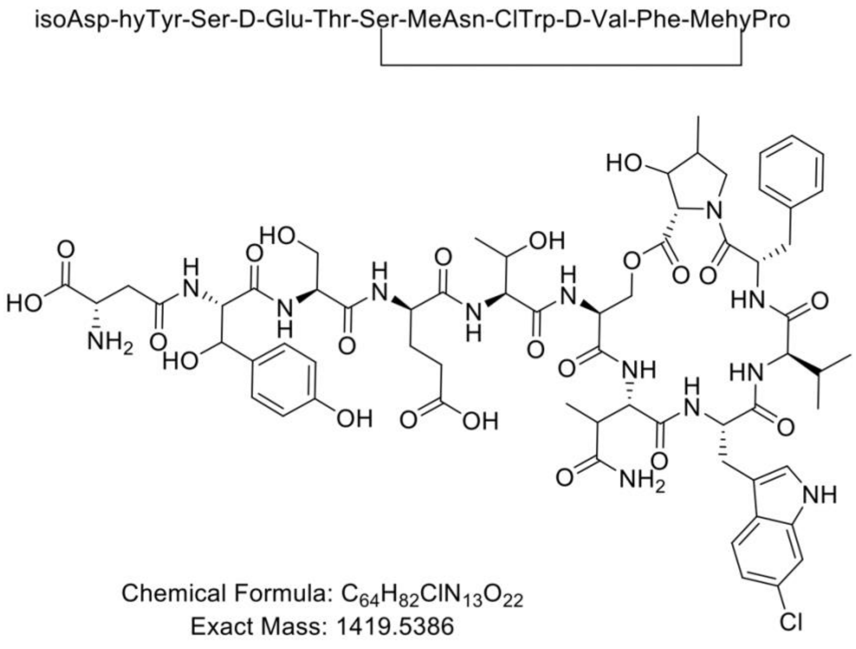

*The stereochemistry of α-protons in amino acid residues were predicted by bioinformatics analysis.

Supplementary Fig. 6 - Chemical structure of omnipeptin **(1)**

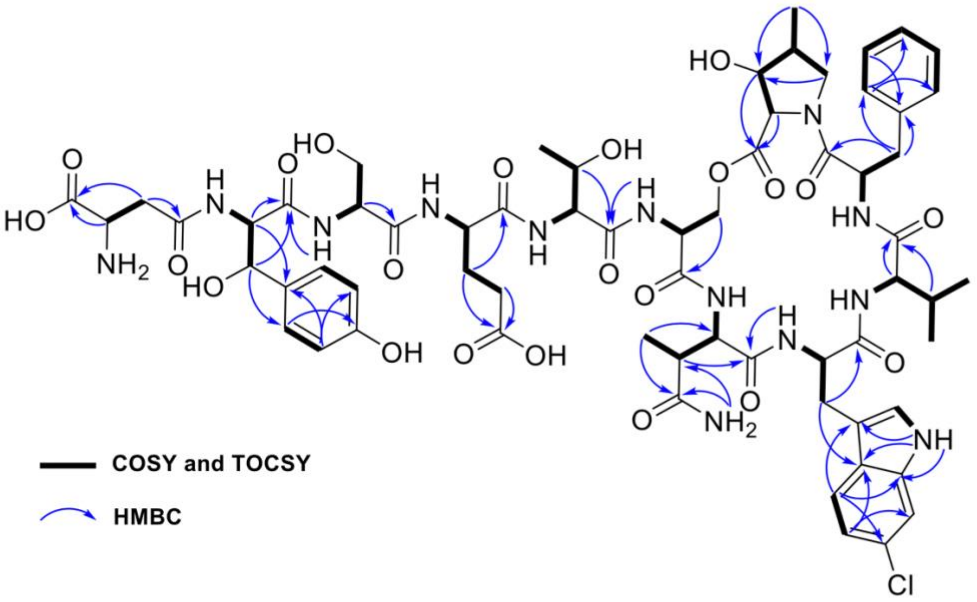

Supplementary Fig. 7 - 2D NMR correlations of omnipeptin (**1**)

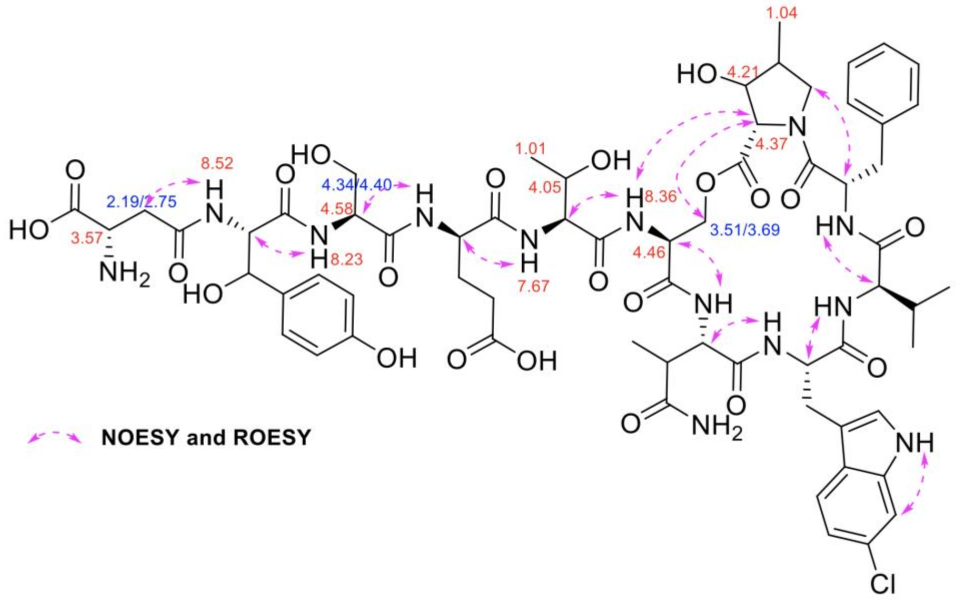

Supplementary Fig. 8 - NOE/ROE correlations of omnipeptin (**1**) establishing the connectivity of amino acid residues

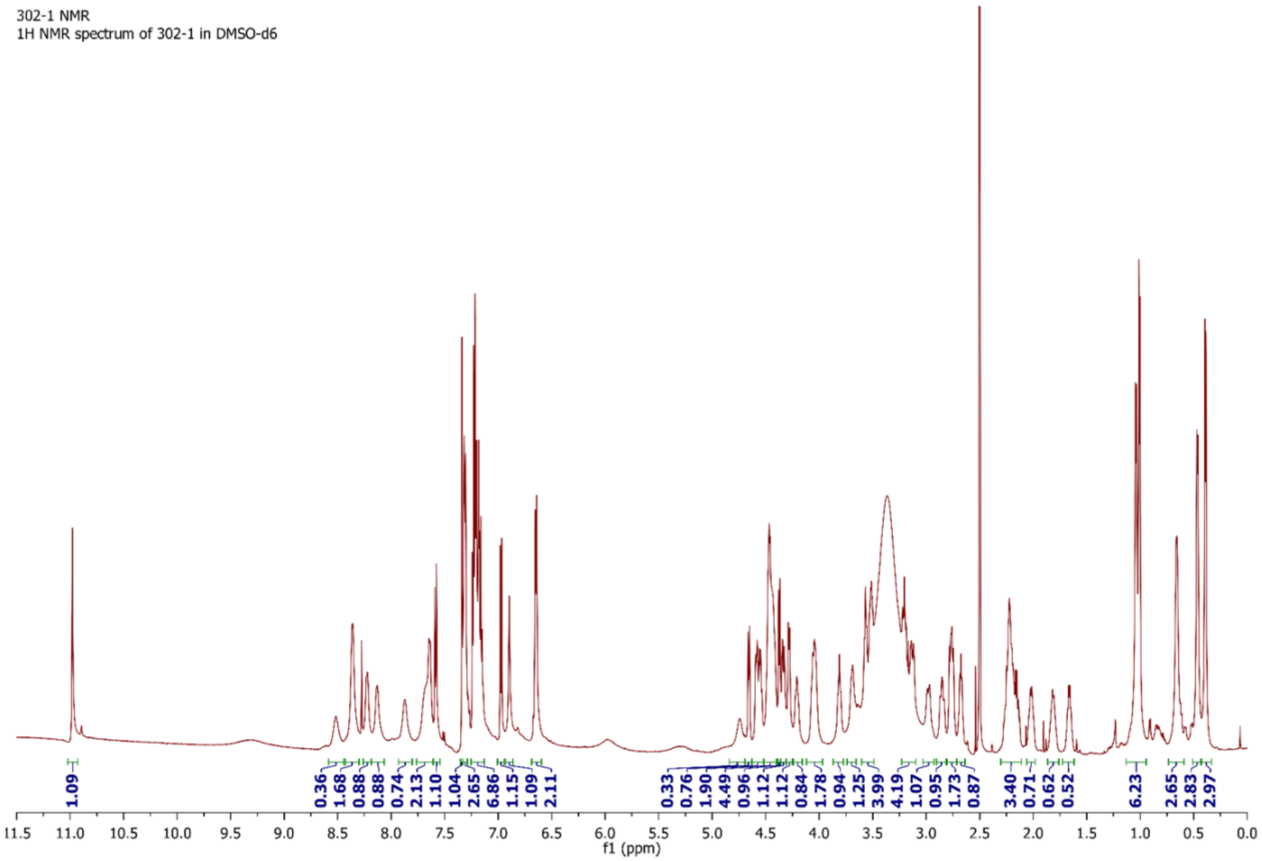

Supplementary Fig. 9 - ^{1}H NMR (600 MHz, DMSO-d_6) spectrum of omnipeptin (**1**)

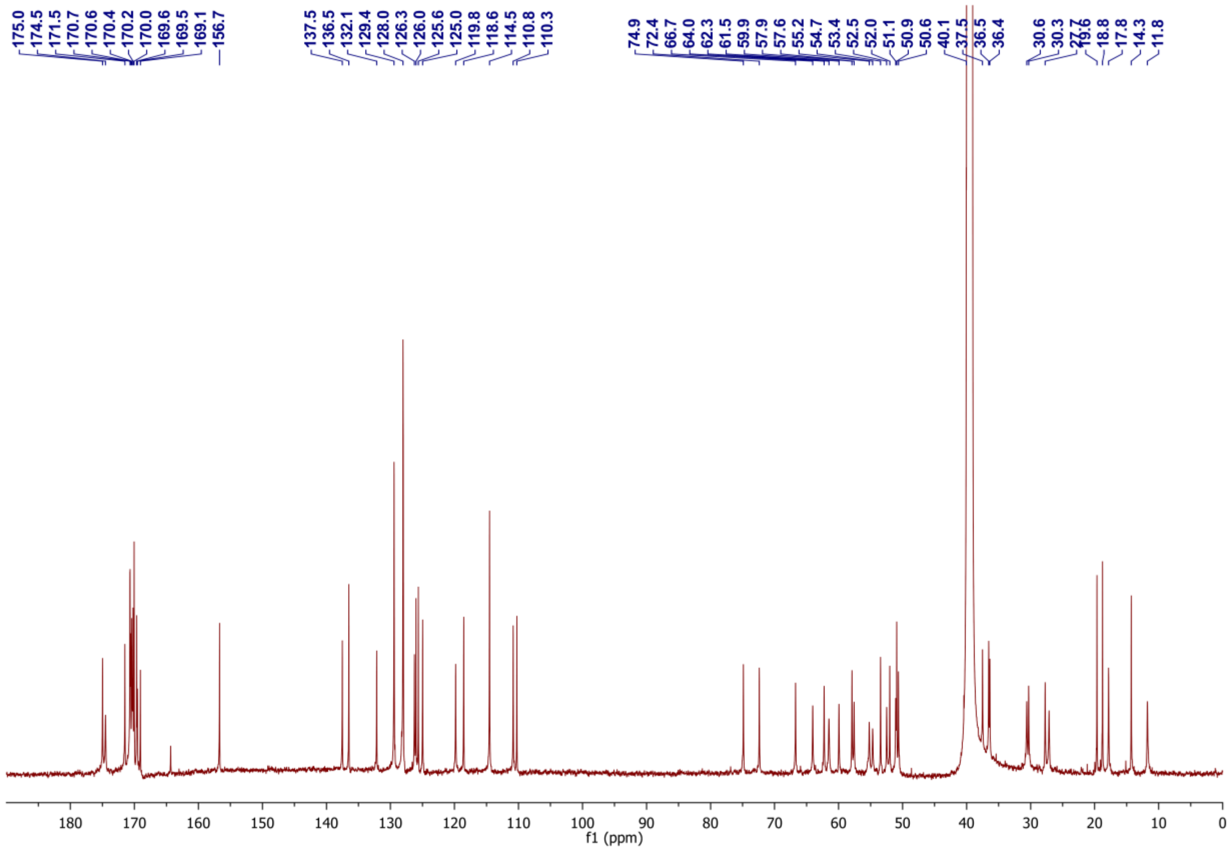

Supplementary Fig. 10 - ^{13}C NMR (150 MHz, DMSO-d_6) spectrum of omnipeptin (**1**)

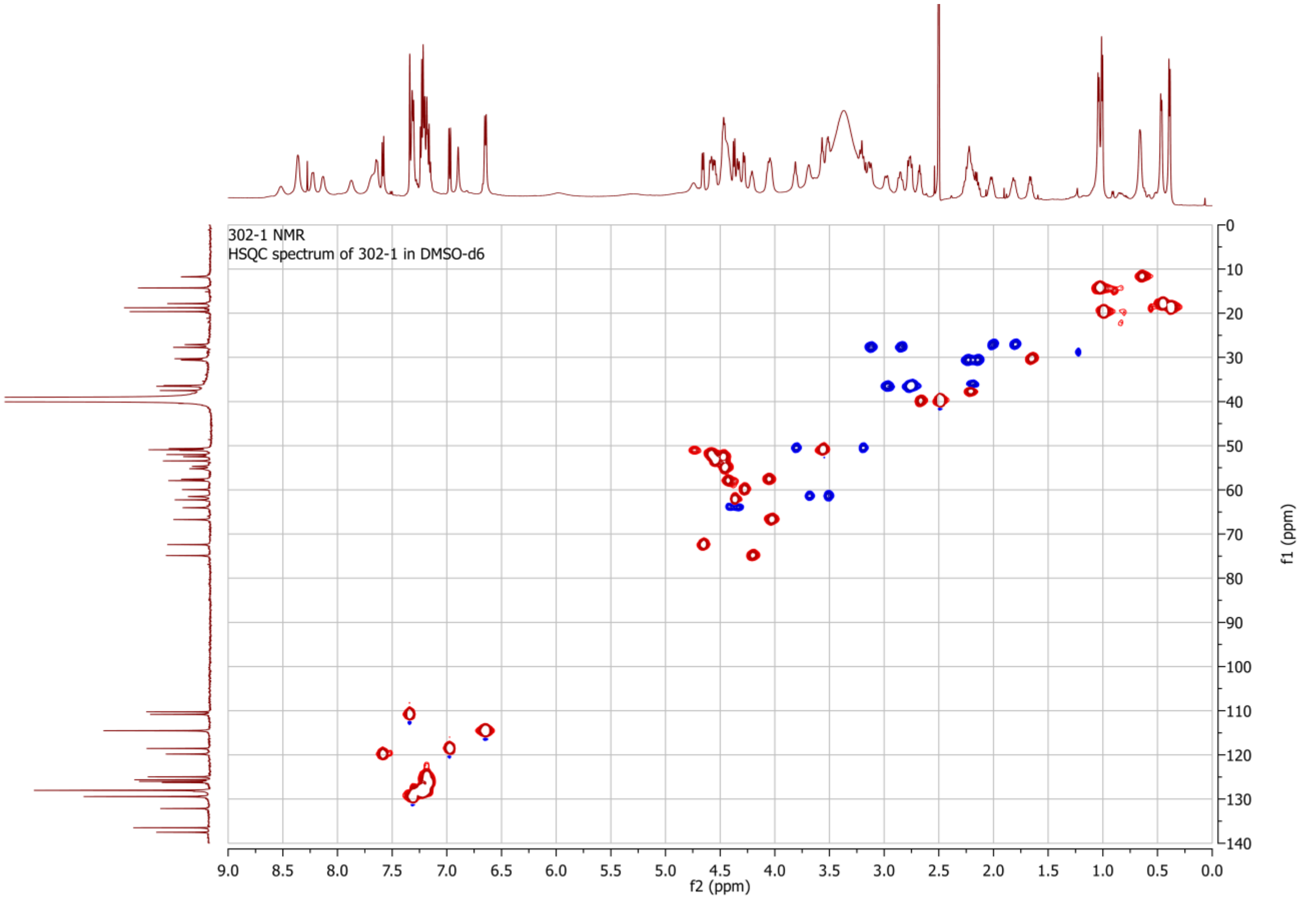

Supplementary Fig. 11 - ^{1}H-^{13}C HSQC NMR (600 MHz, DMSO-d_6) spectrum of omnipeptin (**1**)

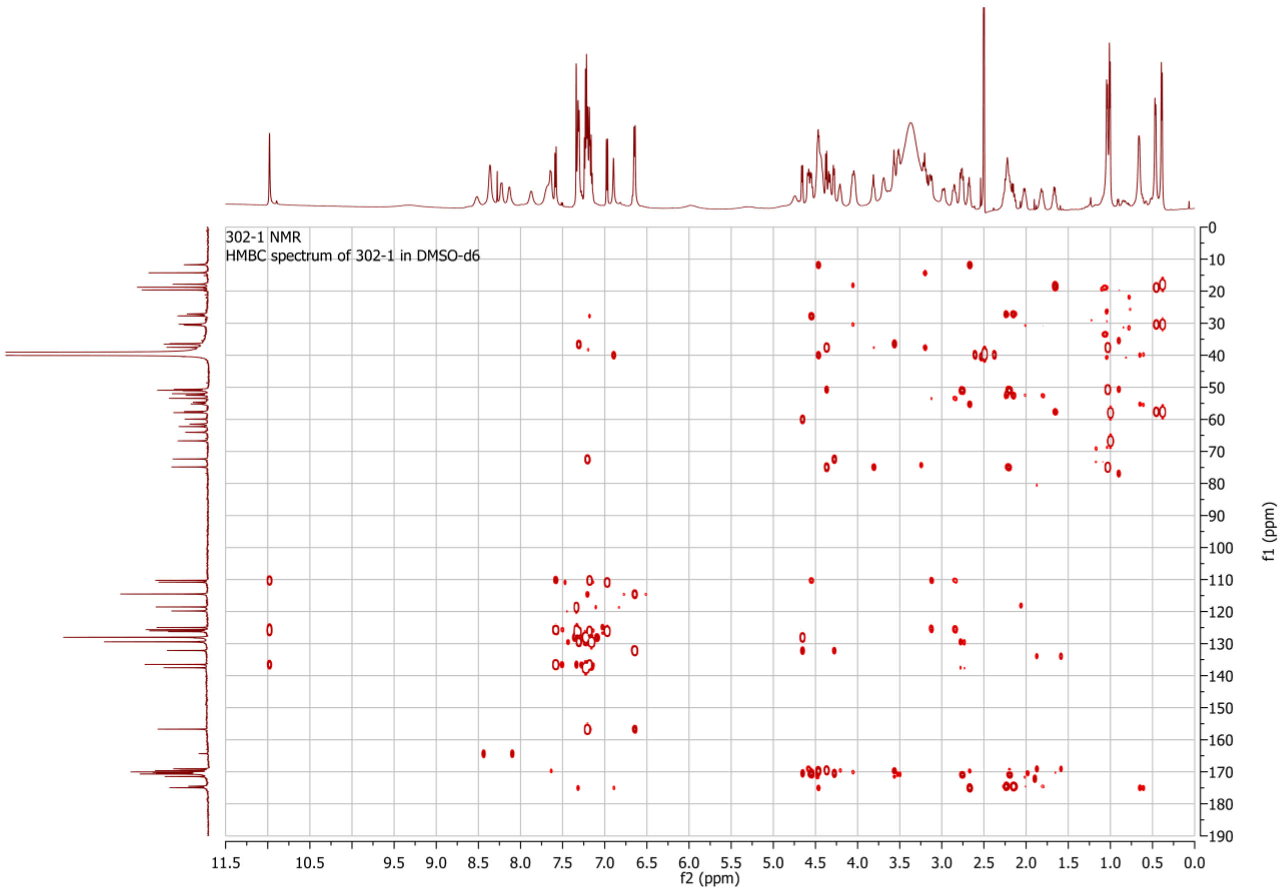

Supplementary Fig. 12 - ^{1}H-^{13}C HMBC NMR (600 MHz, DMSO-d_6) spectrum of omnipeptin (**1**)

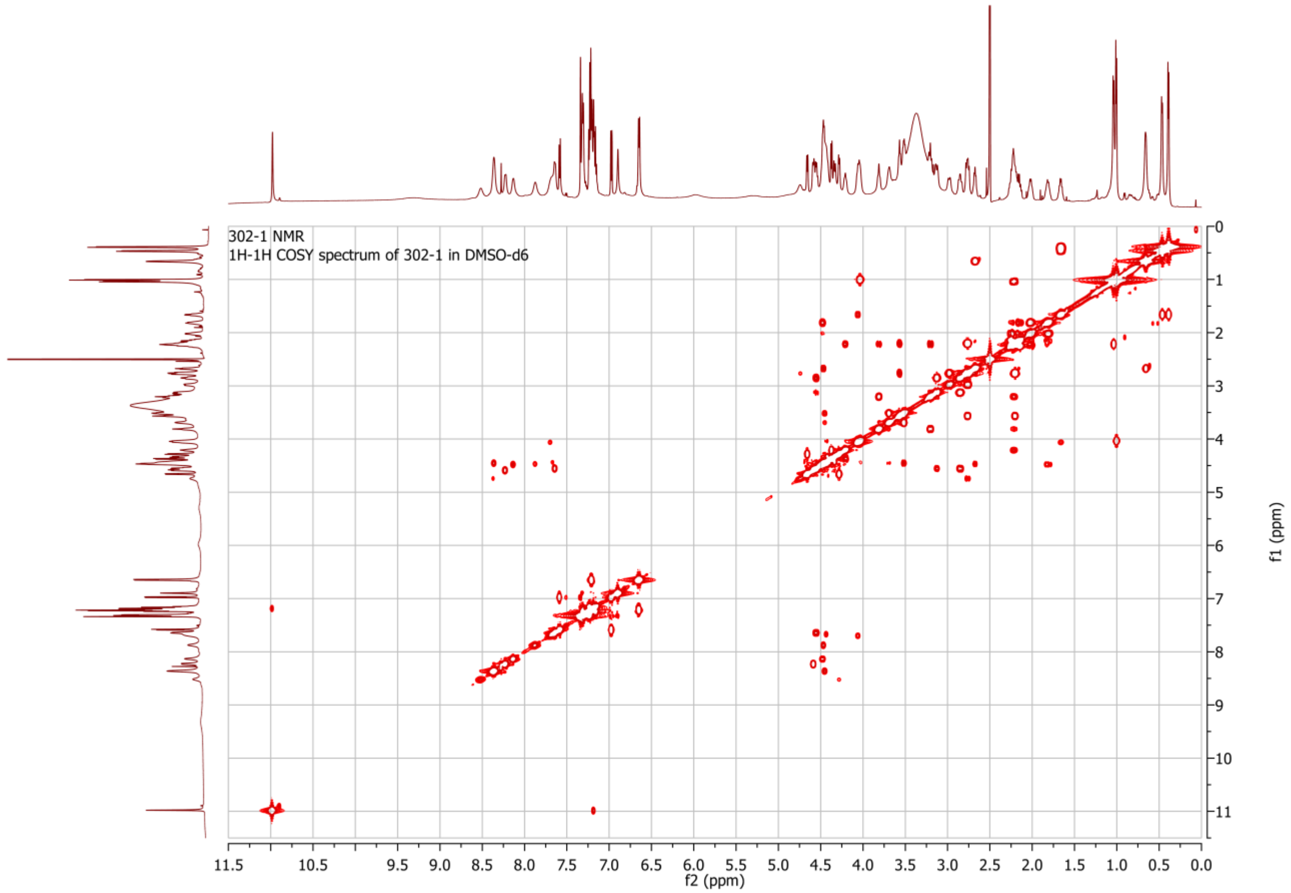

Supplementary Fig. 13 - ^{1}H-^{1}H COSY NMR (600 MHz, DMSO-d_6) spectrum of omnipeptin (**1**)

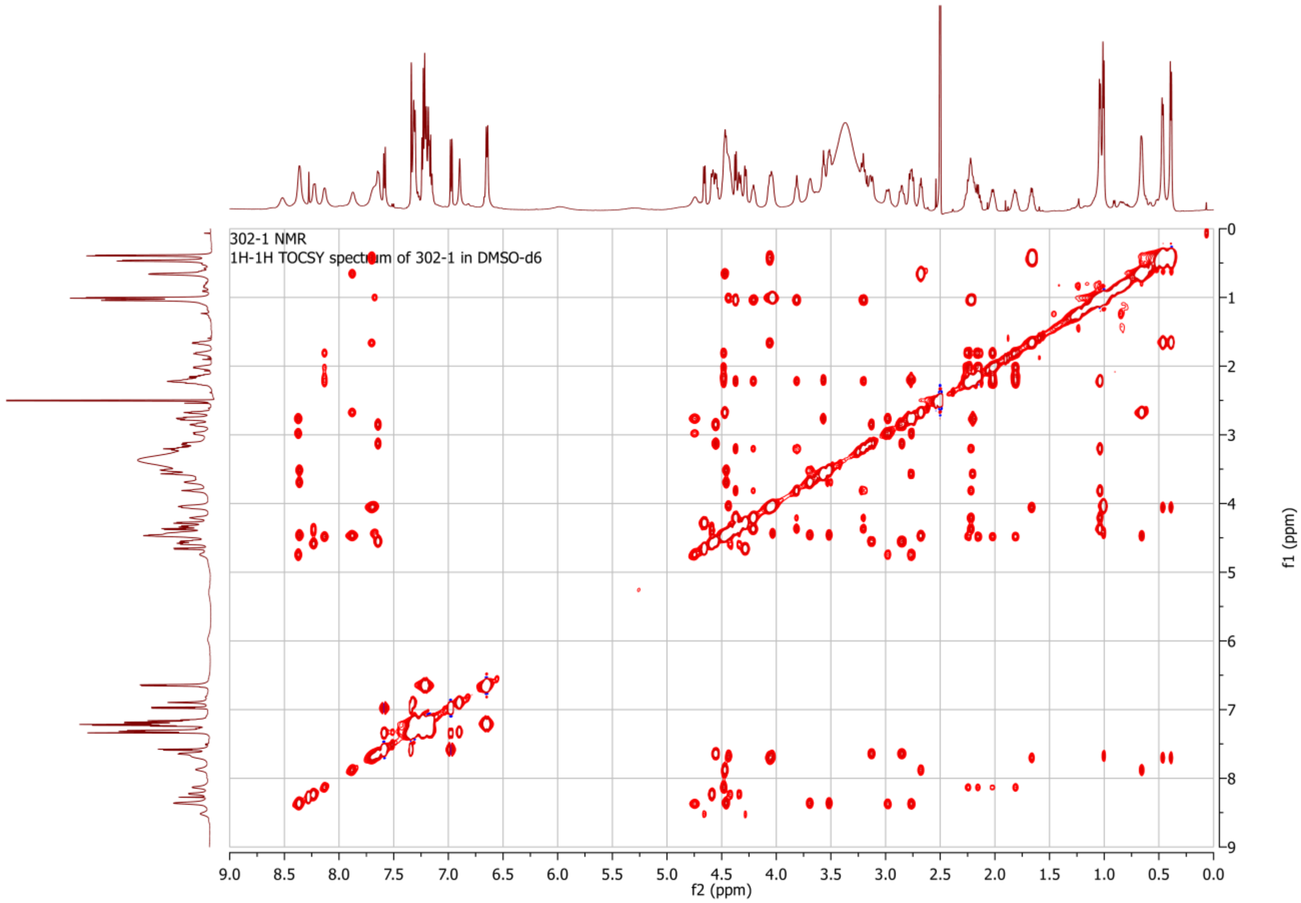

Supplementary Fig. 14 - ^1H-^1H TOCSY NMR (600 MHz, DMSO-d_6) spectrum of omnipeptin (**1**)

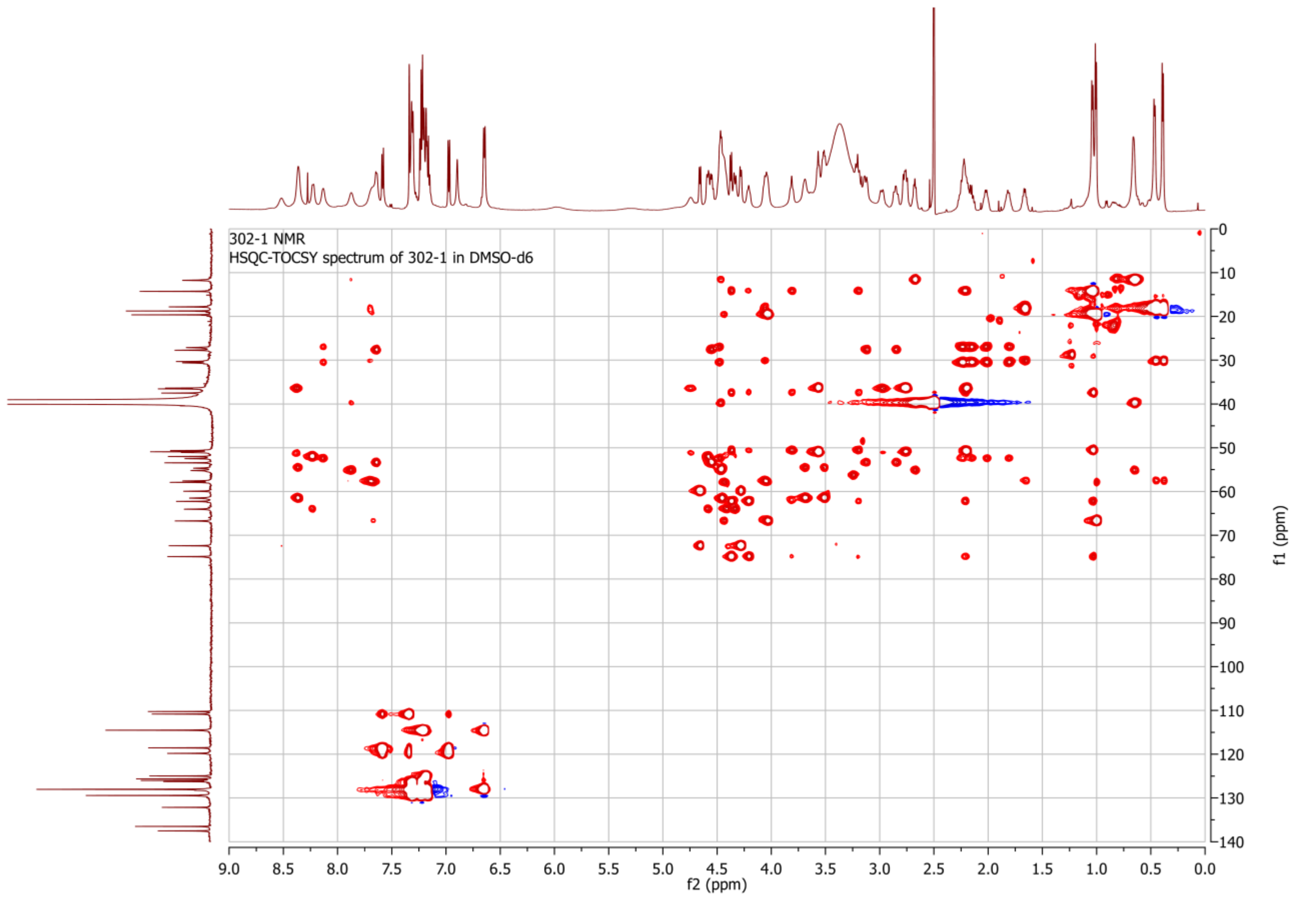

Supplementary Fig. 15 - ^{1}H-^{13}C HSQC-TOCSY NMR (600 MHz, DMSO-d_6) spectrum of omnipeptin (**1**)

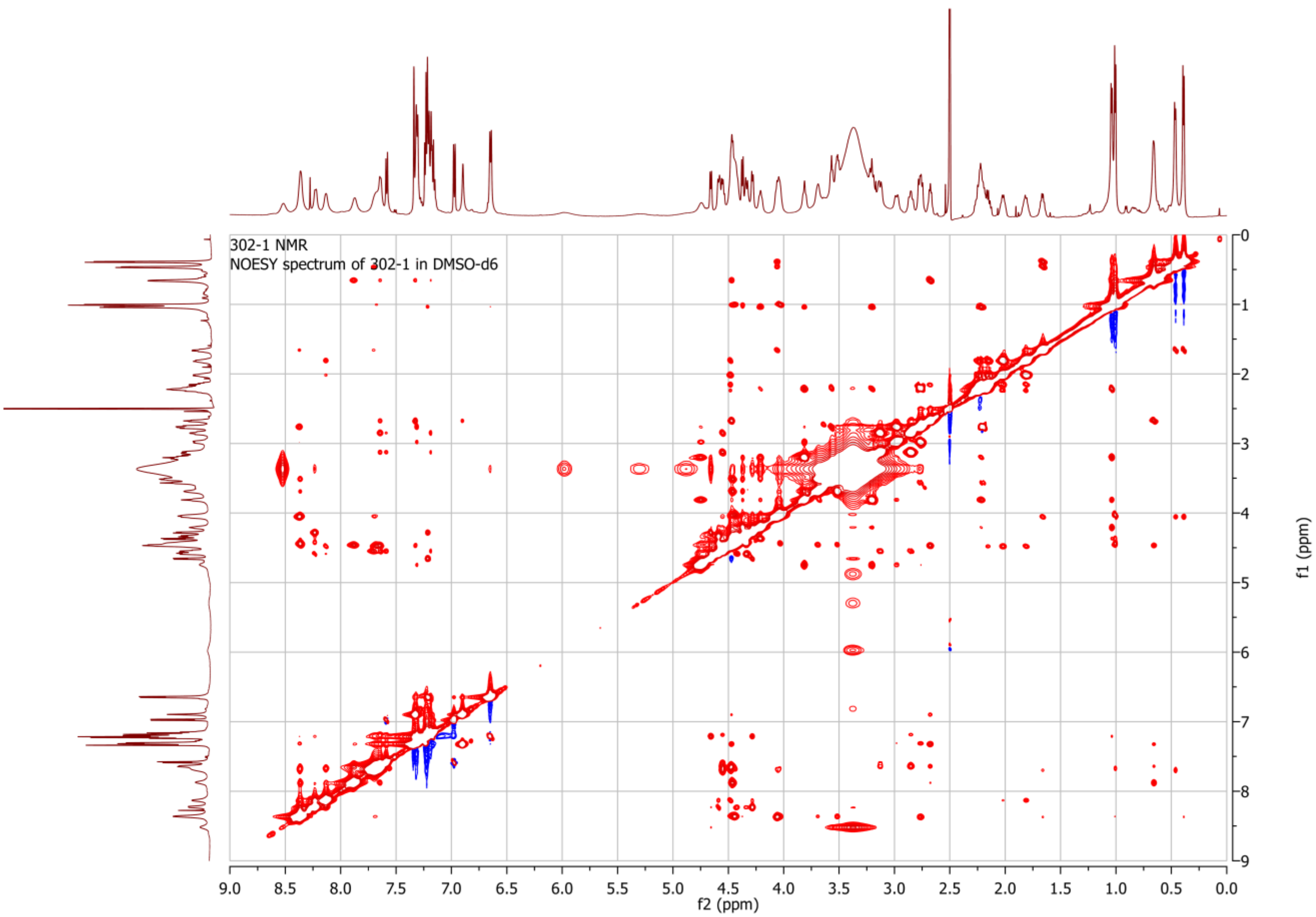

Supplementary Fig. 16 - ^{1}H-^{1}H NOESY NMR (600 MHz, DMSO-d_6) spectrum of omnipeptin (**1**)

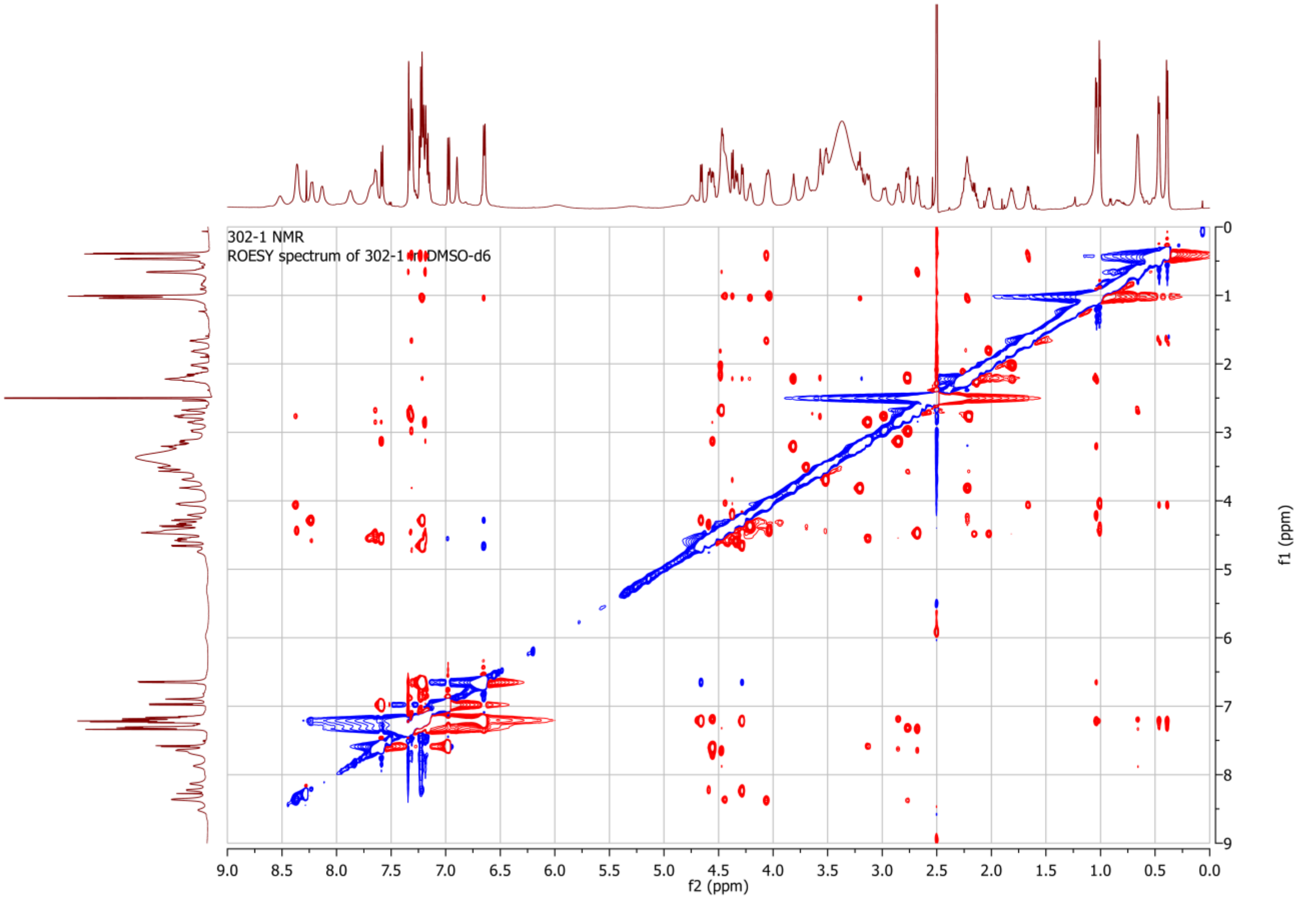

Supplementary Fig. 17 - ^1H-^1H ROESY NMR (600 MHz, DMSO-d_6) spectrum of omnipeptin (**1**)

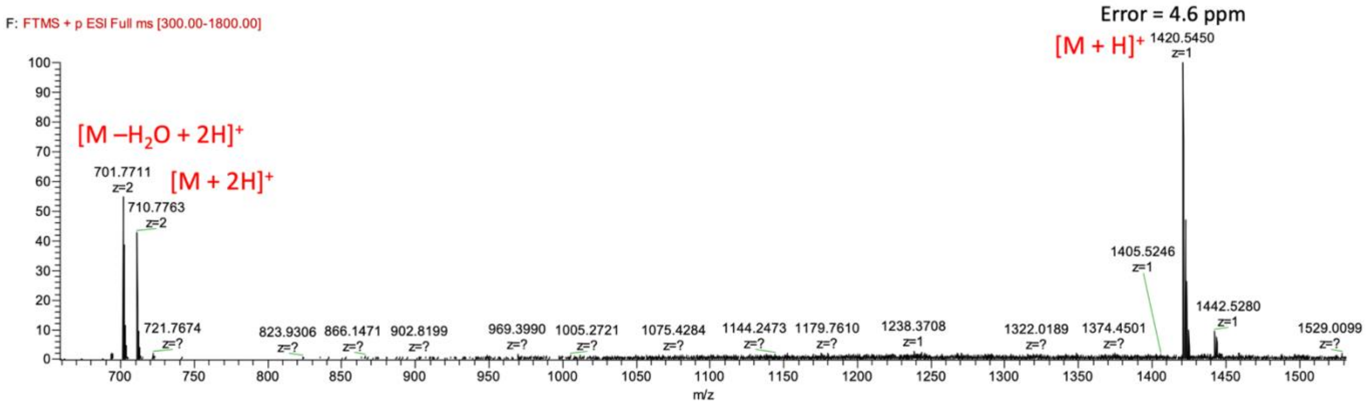

Supplementary Fig. 18 - High-resolution mass spectrometry spectrum of omnipeptin

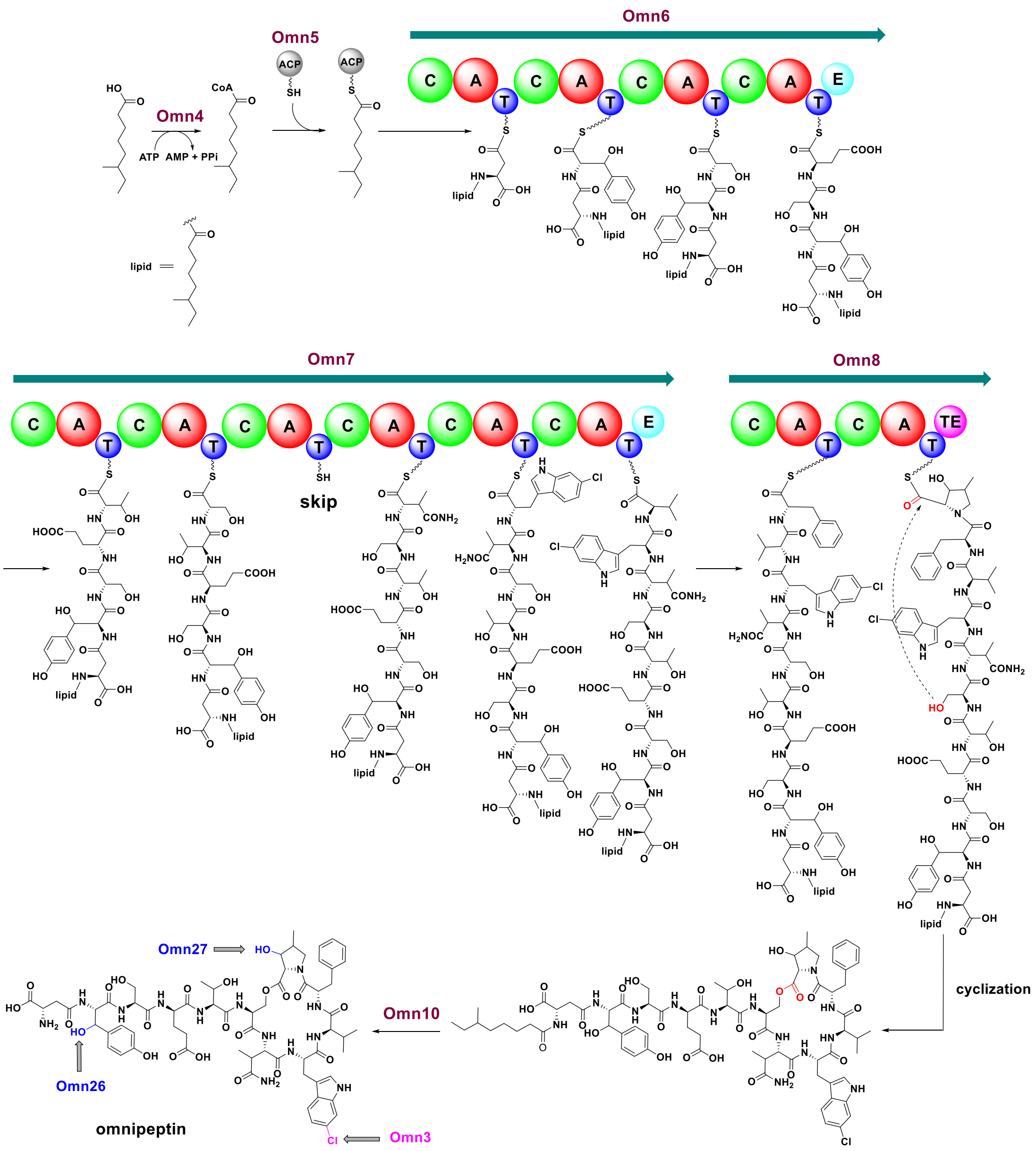

Supplementary Fig. 19 - Proposed biosynthesis of omnipeptin
