# Supplementary material for: Uncovering the biosynthetic potential of rare metagenomic DNA using co-occurrence network analysis of targeted sequences
Source: Nat Commun. 2019 Aug 26;10:3848. doi: 10.1038/s41467-019-11658-z (PMC6710260; doi:10.1038/s41467-019-11658-z)
Supplement: Supplementary file 2 — Description of Additional Supplementary Files [file 41467_2019_11658_MOESM2_ESM.pdf]

Description of Additional Supplementary Files

File Name: Supplementary Data 1
Description: Validation of CONKAT-seq clustering predictions

File Name: Supplementary Data 2
Description: Full sequences of barcoded primers
