# Supplementary material for: Uncovering the biosynthetic potential of rare metagenomic DNA using co-occurrence network analysis of targeted sequences
Source: Nat Commun. 2019 Aug 26;10:3848. doi: 10.1038/s41467-019-11658-z (PMC6710260; doi:10.1038/s41467-019-11658-z)
Supplement: Supplementary file 5 — Reporting Summary [file 41467_2019_11658_MOESM5_ESM.pdf]

nature research

Corresponding author(s): Sean Brady

Last updated by author(s): Jul 18, 2019

Reporting Summary

Nature Research wishes to improve the reproducibility of the work that we publish. This form provides structure for consistency and transparency in reporting. For further information on Nature Research policies, see Authors & Referees and the Editorial Policy Checklist.

Statistics

For all statistical analyses, confirm that the following items are present in the figure legend, table legend, main text, or Methods section.

n/a	Confirmed	
☐	☒	The exact sample size (*n*) for each experimental group/condition, given as a discrete number and unit of measurement
☐	☒	A statement on whether measurements were taken from distinct samples or whether the same sample was measured repeatedly
☐	☒	The statistical test(s) used AND whether they are one- or two-sided<br>*Only common tests should be described solely by name; describe more complex techniques in the Methods section.*
☐	☒	A description of all covariates tested
☐	☒	A description of any assumptions or corrections, such as tests of normality and adjustment for multiple comparisons
☒	☐	A full description of the statistical parameters including central tendency (e.g. means) or other basic estimates (e.g. regression coefficient) AND variation (e.g. standard deviation) or associated estimates of uncertainty (e.g. confidence intervals)
☒	☐	For null hypothesis testing, the test statistic (e.g. *F*, *t*, *r*) with confidence intervals, effect sizes, degrees of freedom and *P* value noted<br>*Give P values as exact values whenever suitable.*
☒	☐	For Bayesian analysis, information on the choice of priors and Markov chain Monte Carlo settings
☒	☐	For hierarchical and complex designs, identification of the appropriate level for tests and full reporting of outcomes
☒	☐	Estimates of effect sizes (e.g. Cohen's *d*, Pearson's *r*), indicating how they were calculated

Our web collection on statistics for biologists contains articles on many of the points above.

Software and code

Policy information about availability of computer code

Data collection	Amplicon sequence data was collected using the MiSeq Control Software (2.6.2.1) on a MiSeq sequencing instrument (Illumina). Untargeted sequencing was collected using the Illumina HiSeq software suite (HCS 2.2.68) on a HiSeq 2500 instrument.
Data analysis	Downstream analysis scripts are publicly available in https://github.com/brady-lab-rockefeller/conkat_seq and https://github.com/brady-lab-rockefeller/BGCs_in_rare_metagenomic_DNA. Analysis was performed using Python 2.7.15 (Anaconda, Inc.) with the following module dependencies: numpy (1.14.3), pandas (0.23.0), networkx (2.1), BioPython (1.68), scipy (1.1.0) and statsmodels (0.9.0). Amplicon sequences were demultiplexed using a Python script avaialbe at https://github.com/brady-lab-rockefeller/paired-end-debarcoder. Primer removal, amplicon length trimming and clustering were performed using VSEARCH (version 2.9.1). Alignment of reads to reference sequences was performed using BBmap (version 38.22), SAMtools (version 1.9), and bedtools (version 2.27.1). Biosynthetic domains were compared to each others and to reference sequences using blastp (version 2.6.0+). Statistical analysis of amplicon co-occurrences was performed using one-sided Fisher's exact test as implemented in the "fisher_exact" function in scipy.stats module. P-values were adjusted to control false-discovery rate using a 2-stage Benjamini-Krieger-Yekutieli procedure as implemented in the "multipletests" function in statsmodels.stats module. Detection and annotation of biosynthetic gene cluster was performed using antiSMASH (version 4.1). Similarity metric scores between biosynthetic gene clusters was calculated using BiG-SCAPE (version 20181005). Domain networks were visualized with Cytoscape (version 3.7.1). NMR data was analyzed with MNOVA.

For manuscripts utilizing custom algorithms or software that are central to the research but not yet described in published literature, software must be made available to editors/reviewers. We strongly encourage code deposition in a community repository (e.g. GitHub). See the Nature Research guidelines for submitting code & software for further information.

Data

Policy information about availability of data

All manuscripts must include a data availability statement. This statement should provide the following information, where applicable:

- Accession codes, unique identifiers, or web links for publicly available datasets
- A list of figures that have associated raw data
- A description of any restrictions on data availability

All sequences of metagenomic inserts harboring BGCs reported in this work are available from genbank (Accession number pending) and from https://github.com/brady-lab-rockefeller/BGCs_in_rare_metagenomic_DNA. Demultiplexed reads of the AD domains and domain networks from the Arizona library are available from https://github.com/brady-lab-rockefeller/BGCs_in_rare_metagenomic_DNA. The remaining data that support the findings of this study are available from the corresponding author upon reasonable request.

Field-specific reporting

Please select the one below that is the best fit for your research. If you are not sure, read the appropriate sections before making your selection.

☒ Life sciences ☐ Behavioural & social sciences ☐ Ecological, evolutionary & environmental sciences

For a reference copy of the document with all sections, see nature.com/documents/nr-reporting-summary-flat.pdf

Life sciences study design

All studies must disclose on these points even when the disclosure is negative.

Sample size	No sample-size calculation was performed
Data exclusions	Sequencing data of ketosynthase domains from one of the six 384 plates of the Arizona library were excluded due to very low sequencing coverage. Consequently only 1920 subpools out of 2304 were processed for Arizona ketosynthase domains co-occurrence analysis.
Replication	n/a
Randomization	n/a
Blinding	n/a

Reporting for specific materials, systems and methods

We require information from authors about some types of materials, experimental systems and methods used in many studies. Here, indicate whether each material, system or method listed is relevant to your study. If you are not sure if a list item applies to your research, read the appropriate section before selecting a response.

Materials & experimental systems

n/a	Involved in the study	
☒	☐	Antibodies
☒	☐	Eukaryotic cell lines
☒	☐	Palaeontology
☒	☐	Animals and other organisms
☒	☐	Human research participants
☒	☐	Clinical data

Methods

n/a	Involved in the study	
☒	☐	ChIP-seq
☒	☐	Flow cytometry
☒	☐	MRI-based neuroimaging
